# Introns mediate post-transcriptional enhancement of nuclear gene expression in the green microalga *Chlamydomonas reinhardtii*

**DOI:** 10.1371/journal.pgen.1008944

**Published:** 2020-07-30

**Authors:** Thomas Baier, Nick Jacobebbinghaus, Alexander Einhaus, Kyle J. Lauersen, Olaf Kruse

**Affiliations:** 1 Bielefeld University, Faculty of Biology, Center for Biotechnology (CeBiTec), Universitätsstrasse, Bielefeld, Germany; 2 Biological and Environmental Sciences and Engineering Division (BESE), King Abdullah University of Science and Technology (KAUST), Thuwal, Kingdom of Saudi Arabia; Washington University School of Medicine, UNITED STATES

## Abstract

Efficient nuclear transgene expression in the green microalga *Chlamydomonas reinhardtii* is generally hindered by low transcription rates. Introns can increase transcript abundance by a process called Intron-Mediated Enhancement (IME) in this alga and has been broadly observed in other eukaryotes. However, the mechanisms of IME in microalgae are poorly understood. Here, we identified 33 native introns from highly expressed genes in *C*. *reinhardtii* selected from transcriptome studies as well as 13 non-native introns. We investigated their IME capacities and probed the mechanism of action by modification of splice sites, internal sequence motifs, and position within transgenes. Several introns were found to elicit strong IME and found to be broadly applicable in different expression constructs. We determined that IME in *C*. *reinhardtii* exclusively occurs from introns within transcribed ORFs regardless of the promoter and is not induced by traditional enhancers of transcription. Our results elucidate some mechanistic details of IME in *C*. *reinhardtii*, which are similar to those observed in higher plants yet underly distinctly different induction processes. Our findings narrow the focus of targets responsible for algal IME and provides evidence that introns are underestimated regulators of *C*. *reinhardtii* nuclear gene expression.

## Author summary

Although many genetic tools and basic transformation strategies exist for the model microalga *Chlamydomonas reinhardtii*, high-level genetic engineering with this organism is hindered by its inherent recalcitrance to foreign gene expression and limited knowledge of responsible expression regulators. In this work, we characterized the dynamics of 33 endogenous and 13 non-native introns and their effect on gene expression as artificial insertions into codon optimized transgenes. We found that introns from different origins have the capacity to increase gene expression rates. Intron-mediated enhancement was observed exclusively when these elements were placed in transcripts but not outside of transcribed mRNA regions. Insertion of different endogenous introns into coding sequences was found to positively affect expression rates through a synergy of additive transcription enhancement and exon length reduction, similar to those natively found in the *C*. *reinhardtii* genome. Our results indicate that intensive mRNA processing plays an underestimated role in the regulation of native gene expression in *C*. *reinhardtii*. In addition to internal sequence motifs, the location of artificially introduced introns greatly affected transgene expression levels. This work is highly valuable to the greater microalgal and synthetic biology research communities and contributes to broadening our understanding of eukaryotic intron-mediated enhancement.

## Introduction

Differential regulation of gene expression is an essential feature of all living organisms that enables adaptation to changing environmental conditions and cellular development. Deciphering the mechanisms of eukaryotic transcriptional regulation has been of interest to the fundamental understanding of genetic processes for many years [1–5]. The effects of *cis*-elements on the core promoter [6] as well as DNA-dependent RNA Polymerase II complex assembly at transcription start sites [7,8] are now well described. A key control mechanism of gene expression is tuning transcriptional activity of the Polymerase II complex, which has now been elucidated in detail [8,9]. Downstream of eukaryotic promoters, gene sequences typically contain introns, non-coding transcript regions which are known to be important elements in evolution as they enable exon shuffling [10] and alternative splicing [11]. In addition, introns have been shown to regulate gene expression [7,12,13] by containing transcriptional enhancers [14] or additional transcription factor binding sites [15], by altering the transcription start site (TSS) [16,17], or by enhancing mRNA stability and export [18]. When incorporated in transgene sequences, introns have also been shown to facilitate higher levels of target expression, a phenomenon called Intron-Mediated Enhancement (IME) which has been observed across many organisms including plants, mammals, insects, and yeast [7,12,19,20]. Apart from basic requirements for IME [12,21], the fundamental mechanisms of this enhancement are poorly understood.

Native gene expression in the unicellular green model microalga *Chlamydomonas reinhardtii* is tightly regulated, which has resulted historically in low transgene expression levels in this host and hindered nuclear engineering strategies. Efforts over the last two decades have combined the application of strong endogenous promoters [22,23] with intensive strain development [24,25] and transgene optimization [26,27] to enhance nuclear transgene expression, each with relative success. With an average of 6.4 introns per gene, the nuclear genome of *C*. *reinhardtii* is relatively intron dense [28]. Alternative splicing of native genes occurs in up to 20% of all transcribed genes [29], compared to 12% in Arabidopsis [30] indicating that intensive mRNA processing is an important factor that likely influences the regulation of gene expression. Previously, the first intron of the *C*. *reinhardtii* ribulose-1,5-bisphosphate carboxylase/oxygenase (RuBisCO) small subunit 2 (RBCS2i1) was found to significantly enhance gene expression [31–33]. It was postulated that the RBCS2i1 contains an intrinsic enhancer element [32,34] but attempts to identify a sequence-related motif within this intron have failed. Other comprehensive investigations of algal introns have not been performed and only a few examples exist where native intron-containing or genomic DNA was applied to mediate overexpression in this host [35–38].

Recently, we developed a sequence optimization strategy which couples effective codon optimization with the systematic insertion of the RBCS2 introns into coding sequences [31]. This transgene design strategy successfully enables reliable nuclear transgene expression in *C*. *reinhardtii* and has now been used to demonstrate several examples of concerted metabolic engineering of this host [39–44]. Despite the broad applicability of the RBCS2i1, the mechanisms of its IME have not been thoroughly investigated and it was unknown if other introns from the algal genome could have similar behaviors.

*C*. *reinhardtii* is a valuable host for the investigation of IME, with relatively short generation times of 6–8 hours, ease of culture and transformation, as well as a growing suite of standardized genetic tools [45,46]. Here, we systematically investigated the IME effect of a large and diverse intron set consisting of 33 endogenous and 13 exogenous intron sequences. Our aim was to identify novel native and non-native introns capable of stimulating IME in this host and to characterize the nature of the IME effect on the efficiency of nuclear heterologous gene expression. For this purpose, the contribution of intron splice sites, deletions and additions of internal sequences, as well as the position effects of IME-stimulating introns in transgene sequences were characterized.

## Results and discussion

### Endogenous introns can stimulate nuclear transgene expression in microalgae

High transcript abundance in the green model microalga *C*. *reinhardtii* cannot exclusively be related to basic promoter activity of the respective gene. This is especially true for highly expressed genes such as those coding for RuBisCO or photosystem subunits, whose promoters are capable of driving transgene expression, but not to the same rate of their endogenous genes. The respective gene sequences likely harbour additional intrinsic features leading to sufficient transcription rates and robust protein accumulation. In addition to regulatory 5’ and 3’ UTR elements, introns are known to play important roles in transcriptional control in many eukaryotic organisms [7,12–18]. Increasing genome and transcriptome data coupled to the ease of DNA synthesis now enables comprehensive investigation of potential candidate intron sequences for their roles in gene expression. In *C*. *reinhardtii*, the capacity of introns to induce IME in intron-containing (trans)genes was initially described for the RBCS2i1 [31–33]. This intron has been found to successfully splice after artificial insertion into transgenes and to reliably elevate rates of transcription [31]. However, the underlying mechanisms and the capacities of other introns to mediate similar effects have not yet been investigated.

**Fig 1:**
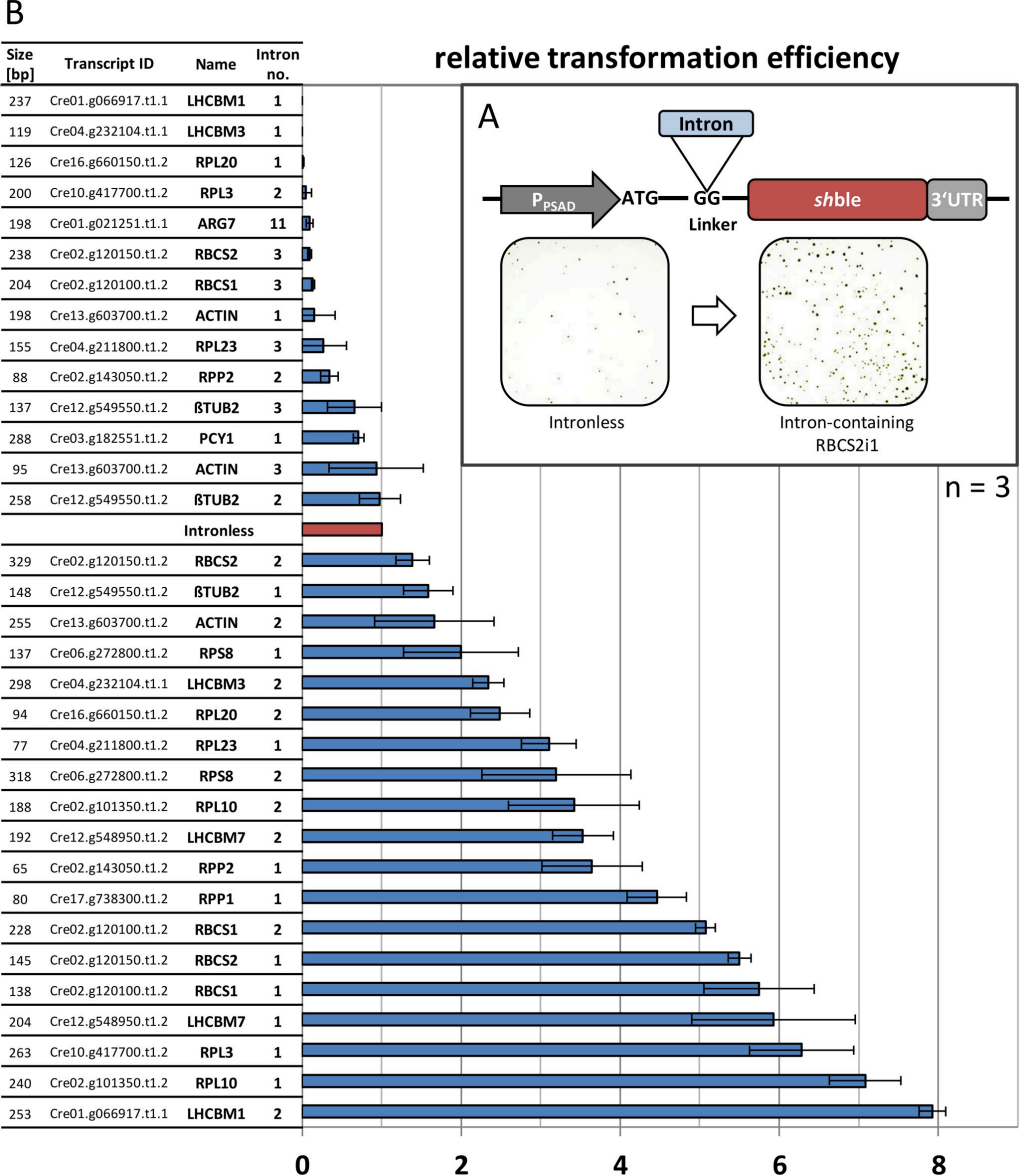
Relative transformation efficiencies of 33 endogenous introns. (A) Gene design of a *sh*ble antibiotic selection cassette for scarless insertions of endogenous introns into a N-terminal linker sequence containing an appropriate splice site (GG). The intron-mediated expression is reflected by the respective number of regenerated transformant colonies normalized to the intronless control. Exemplary transformation plates are depicted for an intronless and an intron-containing RBCS2i1 construct. (B) The respective sequence identity and relative transformation efficiency of investigated endogenous introns. Gene-clustered expression levels are shown in S7 Fig. Error bars represent standard deviations from the mean of triplicate measurements. Complete intron sequences are given in S1 Data. P_PSAD_−promoter and 5’UTR of the *C*. *reinhardtii* PSAD gene, *sh*ble–*S*. *hindustanus* phleomycin resistance gene, 3’UTR– 3′ untranslated region of the *C*. *reinhardtii* RBCS2 gene.

We recently developed a small N-terminal sequence addition to the *sh*ble selection marker to systematically investigate the potential of artificial intron insertions [31]. Functional splicing was indicated directly by the survival of transformants in the presence of zeocin as non- or incorrect splicing results in a frameshift and, consequently, loss of antibiotic resistance [31]. The relative transformation efficiency of this construct directly correlates with the gene expression of the stoichiometric drug-sequestering *sh*ble protein [31,47] (Fig 1A). The capacity to induce IME was investigated for 33 strategically selected candidate introns (S1 Data) originating from 16 native highly expressed genes under standard cultivation conditions identified in the *C*. *reinhardtii* nuclear genome via a transcriptome study [48]. Selected introns were artificially inserted into the N-terminal extension splice site within the *sh*ble expression cassette (Fig 1) and relative transformation efficiency was quantified in comparison to the intronless control construct as an indicator of relative IME effect. Each intron induced a unique, sequence-specific expression pattern ranging from no colonies (frameshifts after impaired splicing), similar or less colonies than the intronless control (inefficient splicing or no effect), or induced IME ranging from subtle improvements up to an 8-fold greater expression compared to the intronless control.

For 3 of the 33 tested introns (9.1%) colonies were never recovered, indicating impaired *sh*ble translation and consequent absence of antibiotic resistance. Eleven of 33 introns (33.3%) resulted in unchanged or impaired expression without complete inhibition of the antibiotic selection, suggesting decreased expression induced by an as of yet undescribed mechanism or inefficient mRNA processing and consequent impaired protein translation [30]. Higher expression was observed for 19 of the 33 analysed introns (57.6%), which indicates that many native *C*. *reinhardtii* introns can stimulate IME, a factor that has been largely overlooked in understanding the regulation of native and transgene expression in this host. We did not observe a clear correlation of intron size and the respective level of IME (Fig 1). The RBCS2i1 was previously identified to effectively stimulate IME, which was confirmed in this study by a 5.5-fold higher expression compared to the intronless control. Several other introns from native highly expressed genes exhibited similar or even higher IME. The strongest increase was observed for the previously undescribed second intron (i2) of the chlorophyll a-b binding protein gene, LHCBM1, which mediated 8-fold higher expression than the control. We did not observe intron retention or alternative TSS usage for four tested introns (RBCS2i1, RBCS2i2, LHCBM1i2, ßTUBi3, S1 Fig) regardless of their levels of IME. This suggests that splicing of artificially introduced introns is generally efficient in *C*. *reinhardtii*, although alternative mRNA processing can occur.

The gradual distribution of expression levels observed across this intron set hinders generalizations of the degree of IME that can be expected from different intron sequences. The first intron (i1) of RBCS2 has been reported across multiple studies to be a reliable and strong IME effecting intron for *C*. *reinhardtii* nuclear transgene expression [31–33,39–43]. In this study, higher IME was observed from the introns natively located in closer proximity to the respective promoter, however, with some exceptions (ACTIN, LHCBM1, RPS8, LHCBM3, and RPL20, S1 Fig). This suggests a conserved interplay between the promoter and intron sequences, which is similar to findings in higher plants [49,50].

As a phototrophic, single-cell eukaryote, *C*. *reinhardtii* is able to tune its gene expression in response to constantly changing environmental conditions, predominantly to adapt to variable light intensity [51,52] and nutrient availability [53]. In addition, some of the analysed introns originate from genes that are involved in light harvesting or photoprotection. To exclude that the applied light regime during selection of transformants has an impact on the *sh*ble expression, we quantified the IME level at different light intensities (S2 Fig). Although recovery on medium light intensities resulted in slightly increased absolute colony counts, likely due to optimal growth conditions, no relative change in IME was observed. Indeed, it rather appears, that IME is involved in constitutive expression [12] and represents a general feature of eukaryotic gene expression regulation.

### Exogenous introns can mediate enhanced gene expression in *C*. *reinhardtii*

**Fig 2:**
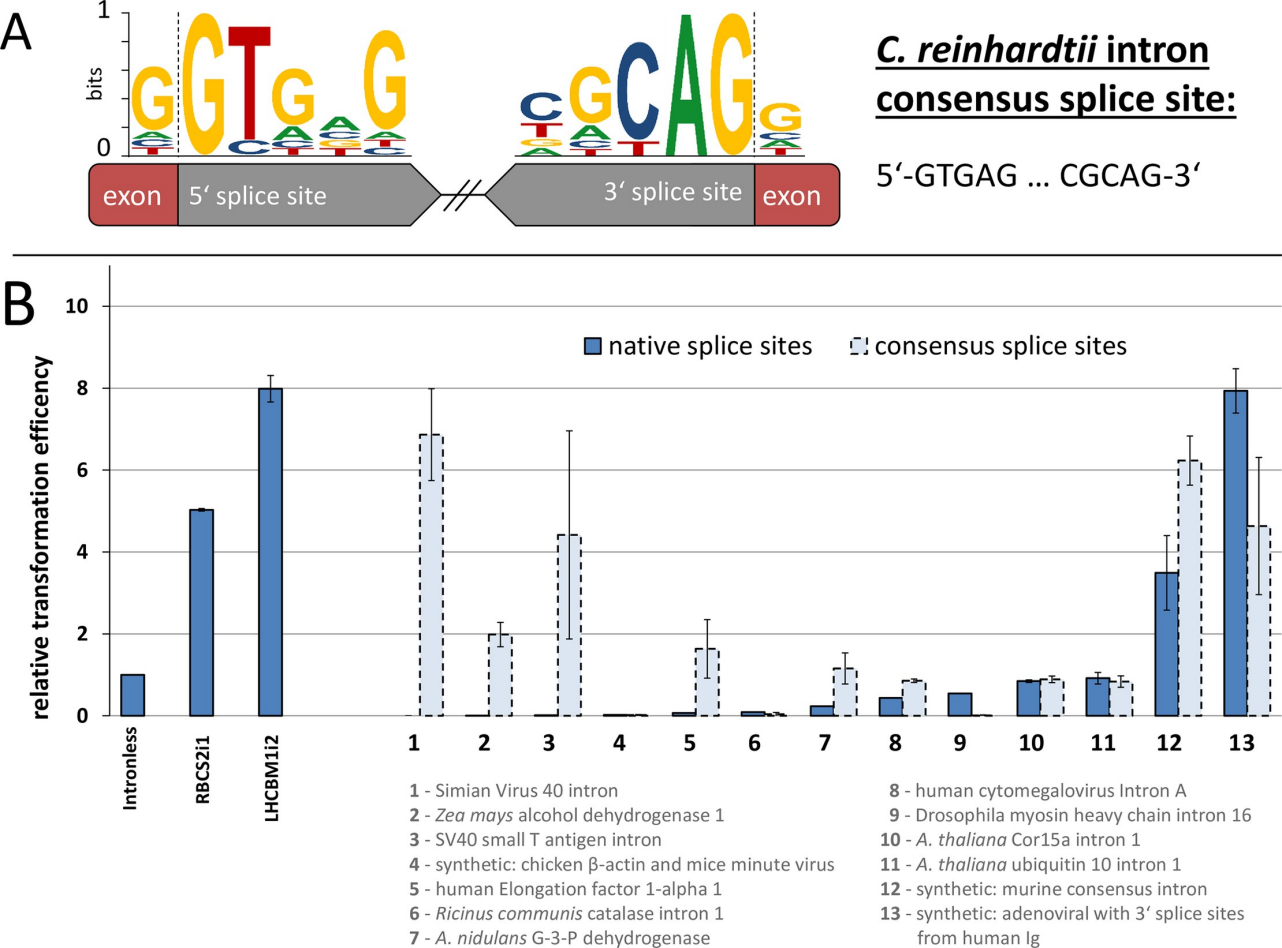
Relative transformation efficiencies of 13 exogenous introns. (A) Schematic Weblogo of intron consensus sequences at the 5’ and 3’ internal splice sites of introns from *C*. *reinhardtii*. (B) The respective sequence identity and relative transformation efficiency of investigated exogenous introns and the endogenous RBCS2i1 and LHCBM1i2 normalized to the intronless control. Efficiency is shown for introns in their native sequence boundaries (dark blue) and after modification to match the *C*. *reinhardtii* consensus sequence (light blue hashed lines). Error bars represent standard deviations from the mean of triplicate measurements. Complete intron sequences are given in S1 Data. P_PSAD_−promoter and 5’UTR of the *C*. *reinhardtii* PSAD gene, *sh*ble–*S*. *hindustanus* phleomycin resistance gene, 3’UTR– 3′ untranslated region of the *C*. *reinhardtii* RBCS2 gene.

Several stimulating introns have been identified in other model systems which originate from viral DNA or different eukaryotic genomes including plants, fungi, and mammals [54–59]. Although their mode of action is not fully elucidated, typically, these introns are applied in genetic engineering strategies as synthetic insertions in transformation constructs or promoters [60]. Here, we selected 13 previously described exogenous candidate introns, including sequences which have been confirmed to induce IME in Arabidopsis [50,61] and tested their capacity to affect expression also in *C*. *reinhardtii* (Fig 2).

The exogenous introns tested caused reduced expression levels compared to the intronless control construct in general. It is likely that the foreign nature of these introns hinders coordination with the algal splicesomal machinery, which is evolved to recognize native internal intron boundaries (Fig 2A). Two exogenous introns were exceptions and caused IME 7.9 and 3.5-fold over the intronless control: an adenoviral intron with internal splice sites from human Ig (Intron 13, Fig 2B) and a synthetic murine consensus intron (Intron 12, Fig 2B), respectively. These introns are derived from evolutionally unrelated mammalian genomes, both to *C*. *reinhardtii* and each other, nevertheless, their sequences clearly induced IME in *C*. *reinhardtii*. The other exogenous introns, mainly derived from the plant kingdom, did not upregulate gene expression (Fig 2B). IME inducing sequences from *A*. *thaliana* UBQ10 which quantitatively enhance gene expression *in planta* [59,62] did not cause IME in *C*. *reinhardtii* (Intron 11, Fig 2B), suggesting different underlying IME signaling regulation mechanisms between plants and green algae. Many nuclear encoded genes in *C*. *reinhardtii* can be traced to the progenitor of both green algae and plants, including genes associated with photosynthesis and plastid function, whereas some are derived from the plant-animal common ancestor [28]. Many of those genes have been lost in angiosperms, notably those encoding proteins of the eukaryotic flagellum and the associated basal body which are still found in *C*. *reinhardtii* [28,63]. It is likely that IME-related mechanisms in Chlamydomonas, and likely other green algae, evolved differently from those found in plants.

The observed low IME levels of exogenous introns led us to consider that the splicing efficiency of non-native internal splice sites may be a limiting factor to successful intron recognition. Indeed, the exogenous intron which exhibited the highest efficiency of IME (Intron 13, Fig 2B) has an internal splice site which matches the algal consensus sequence (Fig 2A) with only a single 3’ nucleotide exchange (G/GTGAG…CACAG/G). This 230 bp long intron derived from an adenovirus substantially improved gene expression in *C*. *reinhardtii*, higher than the well-described RBCS2i1. We postulated that exchanging the respective internal boundaries in non-native introns would contribute to increased transgene expression via adaptation to the host context. After internal splice site optimization, 7 of 13 exogenous introns (53.8%) exhibited improved expression over their non-adapted counterparts with 2 introns exhibiting IME greater than the RBCS2i1 (Intron 1 and 12, Fig 2B). The strongest effect was observed for the simian vacuolating virus 40 intron (SV40, Intron 1, native splice site: G/GTAAG…TCTAG/G), for which splice site adaptation resulted in this otherwise non-splicing intron to exhibit an IME of 7-fold compared to the intronless control. Four introns exhibited no benefit of splice site adaptation (Introns 4, 6, 10, and 11), while two others had hindered efficiencies (Introns 9 and 13). Besides complete intron retention, cryptic splice sites could induce alternative splicing and induce frameshifts which consequently inhibit antibiotic resistance from this construct. The results suggest that effective intron splicing is of great importance for successful transgene expression (Fig 2), however, the individual internal sequences of each intron are responsible for independent IME effects (Fig 1).

### Internal sequence elements responsible for algal IME are dispersed throughout introns

**Fig 3:**
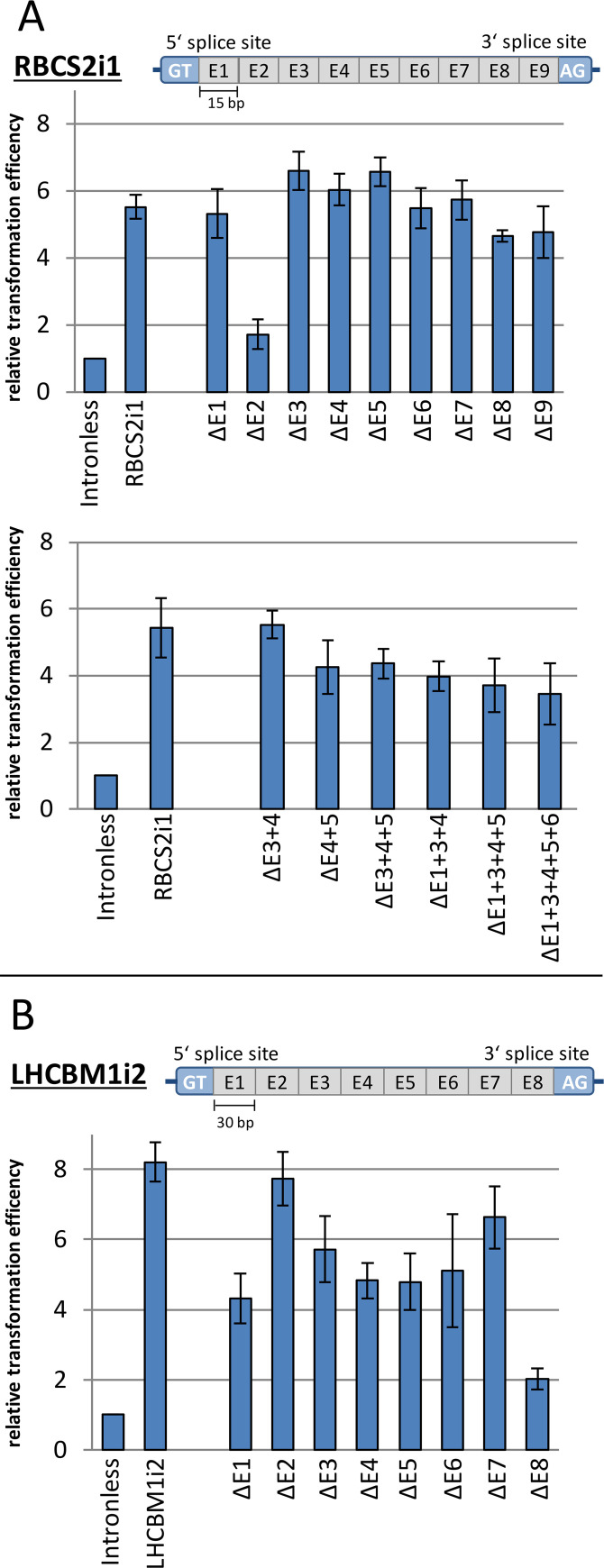
Internal deletion study for two stimulating intron sequences. (A) The RBCS2i1 sequence was subdivided into 15 bp long sequence elements (E1-E9) with 5 bp intron boundaries left un-modified (indicated as GT and AG). The respective relative transformation efficiency after gradual deletion (ΔE1-E9) was compared to the full-length RBCS2i1 and to the intronless *sh*ble control. Below the relative transformation efficiencies are shown after deletions of E1-E9 section combinations from the RBCS2i1 sequence. (B) Corresponding deletion study of LHCBM1i2 with 30 bp deletions (ΔE1-E8). Error bars represent standard deviations from the mean of triplicate measurements.

Given the clear impact of specific intron sequences on IME, we sought to identify any internal sequence motifs that could be responsible for this effect. In order to investigate these internal sequences, a sequential deletion study was performed on the RBCS2i1 and LHCBM1i2 removing individual equal length segments from each and testing their IME efficacy. The native 5 bp internal intron boundaries were retained and the remaining internal sequences were divided into 15 or 30 bp sections for RBCS2i1 and LHCBM1i2 (Fig 3, labelled E1-9 or E1-8, respectively). Variant sequences were generated with internal deletions and the resulting effect on the *sh*ble expression was determined as above.

We observed no severe changes in expression efficiency from removal of E1 and E3-E9 (ΔE1, ΔE3-ΔE9) in RBCS2i1, suggesting that the internal sequences, which can cause IME are dispersed and partially redundant (Fig 3A). Removal of the E2 region (ΔE2) however, resulted in reduced expression. Two scenarios could explain this: deletion of sequence motifs responsible for IME or impaired splicing preventing appropriate *sh*ble expression. To confirm whether this reduction was related to specific IME-causing sequences, the E2 sequence was re-introduced into the RBCS2i1 as two or three copies (RBCS2i1_2xE2 and RBCS2i1_3xE2, respectively, S3 Fig) and into the unrelated exogenous intron 12. Addition of E2 did not alter the IME effect of either intron (S3 Fig), which suggests that the E2 region of RBCS2i1 is likely involved in splice site determination and subsequent processing.

Combinatorial deletions of longer sequence elements (ΔE3+4, Fig 3A) resulted in a reduction of 20% in sequence length (from 145 to 115 bp) without an impact on RBCS2i1 IME. This further suggests that some sequence elements within the RBCS2i1 are redundant and could potentially be removed to lower the nucleotide footprint of this sequence in synthetic intron-containing transgenes for expression in *C*. *reinhardtii*. Further deletions exceeding the E3+4 section resulted in a stepwise reduction of expression (Fig 3A). However, the most truncated version of the RBCS2i1 (ΔE1+3+4+5+6) represents a reduction of 50% in sequence length (145 to 70 bp) but still elicited a 3.8-fold IME over the intronless control.

A similar deletion study was performed for the LHCBM1i2 (Fig 3B). Consecutive removal of 30 bp elements were conducted due to the longer sequence length of this intron (253 bp) and reduced expression was observed for every section tested (ΔE1-E8). These longer deletions resulted in more severe IME reductions similar to the 30 bp deletion in RBCS2i1 (ΔE4+5, Fig 3A). The largest impact on LHCBM1i2 IME was observed for the deletions of E1 and E8 (48% and 75% reduction, respectively) and is likely due to interference with splicing mechanics, as these deletions are directly adjacent to the intron boundaries. Individual deletions of E2-E7 differed in their respective lower IME than full-length (6–41% respectively) but no deletion abolished IME completely.

For both introns, we did not identify any specific internal effector sequences to be responsible for their IME. These findings strongly suggest that enhancement is related to secondary sequence properties that are dispersed throughout the intron and are likely different than the distinct motifs known for enhancers or other DNA-binding proteins (e.g. transcriptions factors, restriction enzymes, recombinases). The absence of a distinct enhancer sequence motif is supported by a lack of conserved sequence domains in a multiple sequence alignment using the 6 highest IME inducing introns found in this work (S4 Fig, Clustal Omega [64]). Also a motif-based sequence analysis did not result in statistically significant predictions of potential consensus motifs when introns with a clear increase in IME of 2-fold or higher were used (S5 and S6 Figs, MEME [65]).

### RBCS2i1 does not contain a classical enhancer and IME occurs exclusively post-transcriptionally

**Fig 4:**
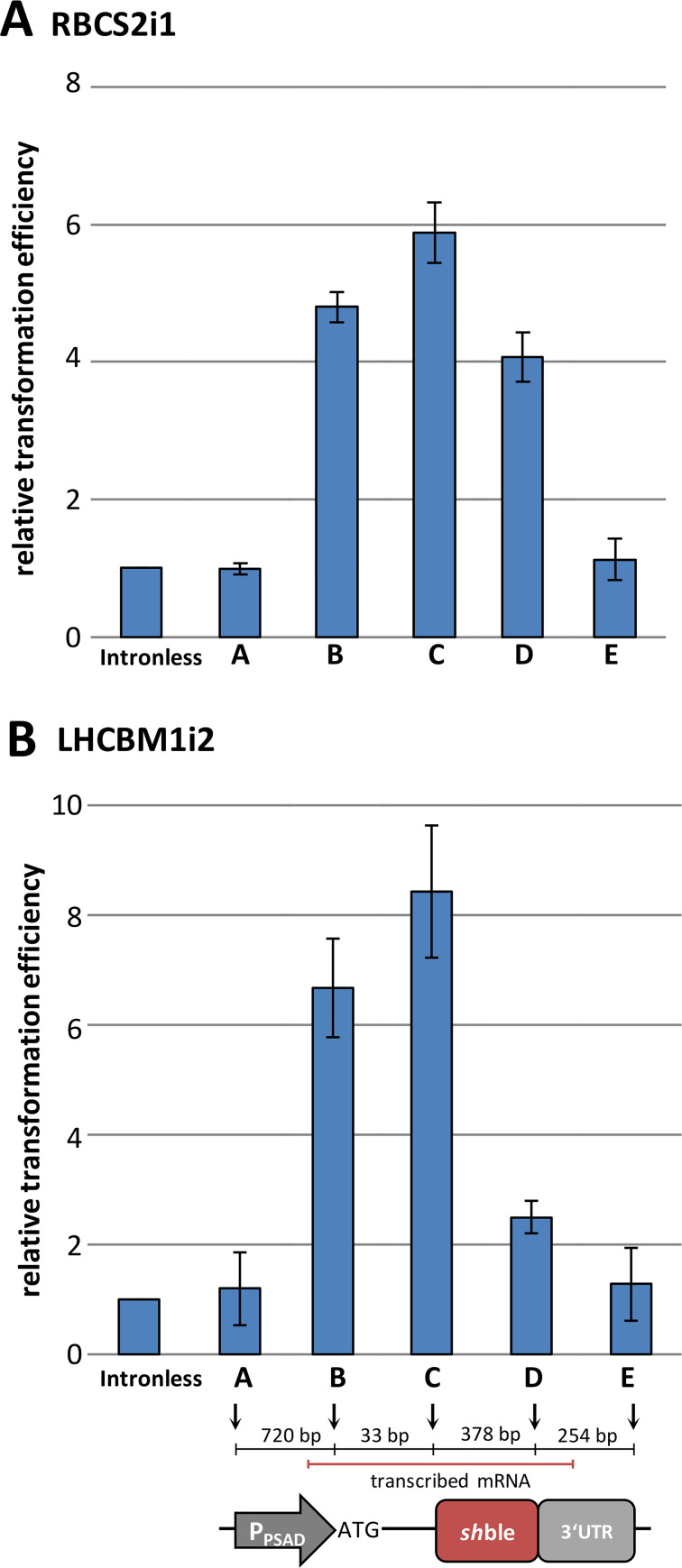
Relative transformation efficiency of the *sh*ble construct containing RBCS2i1 and LHCBM1i2 insertions at different positions. Insertion sites were selected based on the gene design and availability of respective restriction enzyme recognition sites present in the vector system. The insertion positions and the respective distances are indicated in a schematic figure: A—in front of the PSAD promoter (MluI), B—in the 5’UTR (HindIII), C—in the CDS, near the TSS (SmaI), D–in the 3’UTR (XhoI), E–downstream of the construct (KpnI). Error bars represent standard deviations from the mean of triplicate measurements. P_PSAD_−promoter and 5’UTR of the *C*. *reinhardtii* PSAD gene, *sh*ble–*S*. *hindustanus* phleomycin resistance gene, 3’UTR– 3′ untranslated region of the *C*. *reinhardtii* RBCS2 gene.

Classical enhancers of eukaryotic transcription harbor distinct DNA-binding motifs and are known to affect gene expression (after DNA-bending) even from a far distance in relation to the transcription start site (TSS). In contrast, IME is exclusively observed post-transcriptionally, and the intron position within target transgenes is an important factor in the level of enhancement [7,12,66]. Previous findings postulated that enhanced expression mediated by RBCS2i1 also occurs when it is located upstream of the promoter [32,67] and that the sequence likely contains a classical enhancer [32,34]. Therefore, we tested several integration positions of RBCS2i1 and LHCBM1i2 in the *sh*ble screening construct (Fig 4). Position A and E are defined as upstream and downstream of the ORF and are not part of the transcript. Compared to the intronless control, the insertion of either RBCS2i1 or LHCBM1i2 clearly did not alter the expression when placed outside of the transcribed region. Although this is in contrast to previous findings for the RBCS2i1 [32,67] in *C*. *reinhardtii*, it is similar to findings of IME-inducing introns in plants [7,12,21]. Even though the underlying mechanism remains elusive, our results indicate that stimulating introns have to undergo transcription and successful splicing to effectively induce IME in *C*. *reinhardtii*.

Strong, endogenous promoters in *C*. *reinhardtii* were previously designed to contain the RBCS2i1 in the corresponding 5’UTRs [22,67,68]. After insertion of stimulating introns into position B within the *sh*ble construct (Fig 4), strong IME was observed for the RBCS2i1 and LHCBM1i2, with 4.8-fold and 6.7-fold higher gene expression, respectively. The PSAD promoter used in this study natively drives expression of an intronless gene [69]. Although, specific acceptor sequences for intron-mediated inducers are likely not found in the PSAD promoter, downstream introduction of stimulating introns improved expression. This finding indicates IME signaling could be interacting with a promoter core element universally conserved in *C*. *reinhardtii* or that the mechanism is promoter independent.

In Arabidopsis, an alteration of the TSS has been observed for stimulating intron insertions near the promoter [17]. We did not observe alternative TSS when several stimulating or non-stimulating introns were placed into the *sh*ble expression construct (Position B: 33bp downstream of the PSAD TSS (S1 Fig)), further suggesting differences in the regulation of IME between green microalgae and higher plants.

Higher IME rates were generally observed for introns natively closer to promoters for endogenous genes (Fig 1, S7 Fig). Integration positions C and D (Fig 4) represent different distances to the TSS in the PSAD (S2 Data). Stimulating introns in close vicinity to the TSS (Position C, 71 bp downstream of the TSS) showed strong IME signals, with an 8.4-fold increase after LHCBM1i2 insertion, whereas in position D (467 bp downstream) insertion resulted only in a two-fold increase. This observation hints to a post-transcriptional influence between splicing of stimulating introns and the consequent rate of transcription, exhibiting two potential IME-related triggers: gene expression could be stimulated by the excised, catalytically active intron RNA or alternatively, successful spliceosome activity stimulates enhanced gene expression by a yet unknown feedback mechanism.

**Fig 5:**
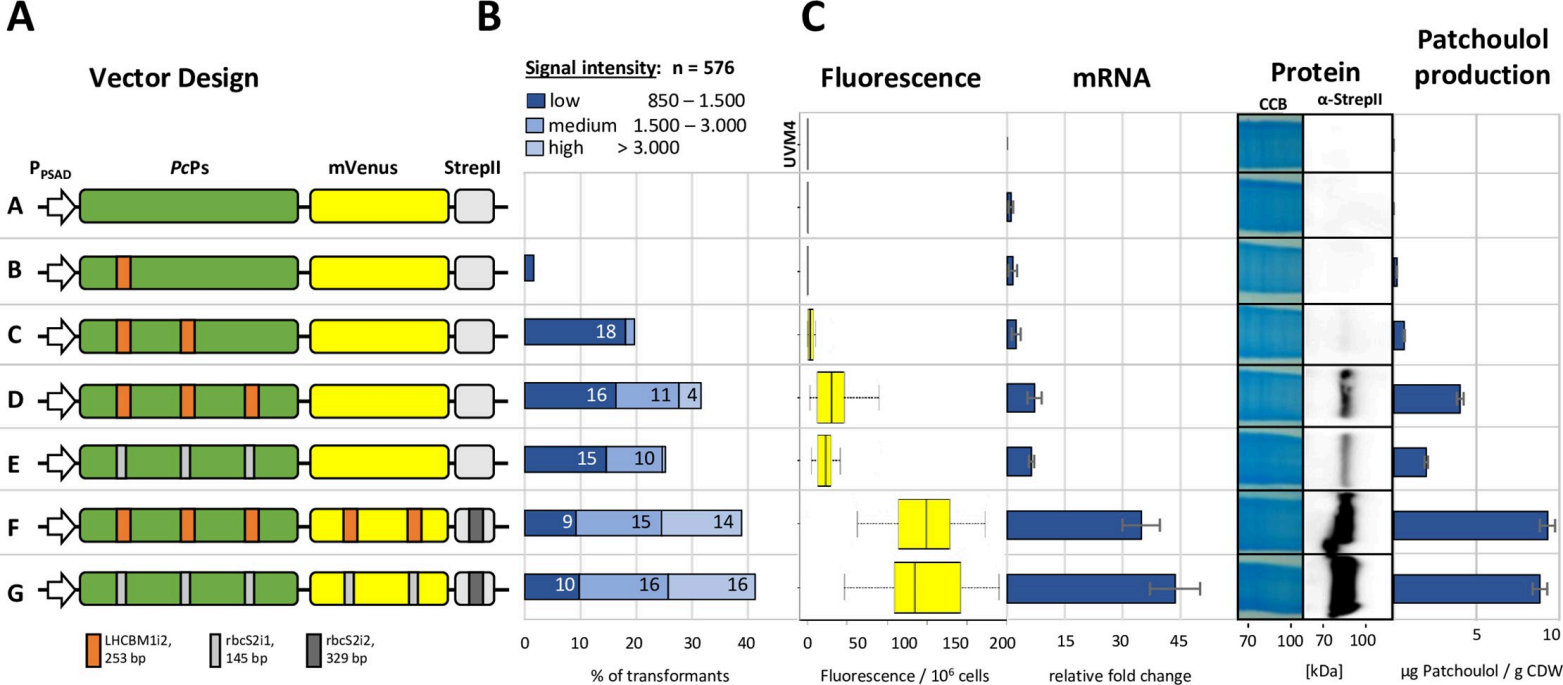
Effects of intron addition on transgene expression levels of the codon-optimized *P*. *cablin* Benth. patchoulol synthase (*Pc*Ps) gene. (A) Vector design of intron-containing construct variants in the modified pOptimized vector (vector A-G, sequence S3 Data). (B) Initial fluorescence screening of 576 isolated transformants. Absolute signals were grouped into three intensity levels: low (850–1500 relative fluorescence units, rfu), medium (1500–3000 rfu) and high (>3000 rfu). Numbers indicate the respective percentage of the initial population. (C) The 20 best transformants from the initial population were isolated and expression was quantified as mean fluorescence per cell, relative mRNA abundance (RTqPCR), protein titer (WB–western blot, α-Strep-tag II with Coomassie Brilliant Blue (CBB) as loading control) and patchoulol production (GC-MS). Cultivations were conducted in microtiter plates and transformants were pooled according to their respective cell densities prior to analysis. Samples of strain UVM4 served as the respective parental control. Error bars represent standard deviations from the mean of triplicate measurements of pooled 20 transformants. P_PSAD_−promoter and 5’UTR of the *C*. *reinhardtii* PSAD gene, mVenus–yellow fluorescent protein variant, StrepII–Strep-tag II epitope.

Splicing usually takes place co- or post-transcriptionally within minutes after transcription initiation [70], where introns are rapidly excised and mature mRNA is exported. Recently, it was shown that instead of undergoing subsequent nucleotide recycling, excised introns can remain active as regulatory RNA (e.g. miRNA [71], snoRNA [72] or lncRNA [73,74]) and effectively alter gene expression. In addition, splicing-associated signalling was recently described in yeast, where polymerase pausing is imposed upon transcription of an intron sequence, inducing a transient polymerase arrest in close proximity to the intron 3′ end until successful splicing occurs [75]. It is therefore possible that spliced nucleotides act catalytically to enhance their host-specific gene expression, for example as an RNA-based elongation factor during transcription. Although this would be in agreement with a post-transcriptional nature of IME, we found incredible variability in expression intensity observed across different introns which was not correlated to sequence length or conserved internal sequences (Fig 1). Our observations support the possibility of specific sequence-related tuning which further complicates interpretation. In IME however, higher relative mRNA abundance can be observed upon successful splicing compared to the intronless control, or those introns which exhibited reduced *sh*ble antibiotic resistance efficiencies (Fig 5)[7,31]. Splicesomal activity could enact feedback signalling to maintain active, locus-specific transcription, leading to higher mRNA accumulation, although the mechanistic details are currently unknown.

Induced signalling during IME could result in a higher degree of DNA accessibility around the start of transcription initiation. This can be achieved by nucleosome shuffling or histone modifications, although this is likely not the primary determinant of gene regulation [76]. In addition, structural organization and architecture of nuclear DNA contributes to the efficiency of transcription and subsequent processing [77]. It was shown that introns can cause a physical interaction of the promoter and the terminator regions by DNA looping [19]. In this scenario, transcription termination leads to immediate re-initiation of the active Polymerase II-complex, inducing higher transcription rates and an elevated mRNA accumulation. Although clear IME phenotypes have been observed in this study, further experiments are necessary to gain deeper insights into the underlying mechanisms of IME in microalgae.

### Intron-containing algal transgenes enable successful genetic engineering strategies

We have previously demonstrated that repetitive insertions of RBCS2i1 can be used to enable robust expression of large heterologous transgenes from the nuclear genome of *C*. *reinhardtii* [31]. Insertions are thought to mimic the host nuclear genome regulation by minimizing exon length in combination with activated IME [31,78]. This gene design strategy has now been frequently used for biotechnological studies aimed at metabolic engineering and recombinant protein expression in *C*. *reinhardtii* [31, 39–44, 79]. Here, we identified several intron sequences that induce strong IME comparable or even higher than RBCS2i1 (Fig 1). An expanded suite of introns for use in transgene design would also help avoid repetitive sequence use, assist in gene synthesis efforts, and could reduce potential unwanted recombination events. Therefore, we chose to determine whether the LHCBM1i2, as the best performing intron in our studies, could also be applied for transgene optimization.

The 1,662 bp, codon-optimized *Pogostemon cablin* Patchoulol synthase (*Pc*Ps) is poorly expressed without introns from the nuclear genome of *C*. *reinhardtii* and has been a reliable gene expression reporter due to its size and linear correlation between the protein abundance and production of the plant secondary metabolite patchoulol (Fig 5, [31]). Suitable splice sites were identified throughout the CDS and used for stepwise insertion of the LHCBM1i2 and RBCS2i1 as previously described (Fig 5A)[31]. Initial expression based on absolute fluorescence intensity at the agar plate level was recorded individually for 576 randomly isolated transformants per construct (Fig 5B). Expression quantification of the recombinant constructs were subsequently performed for all cellular levels including mRNA abundance, fluorescence intensity, protein accumulation and product formation to determine the effect of IME for each intron insertion (Fig 5C). Due to non-homologous end-joining of exogenous transgene DNA into the nuclear genome, individual transformants contain different chromosomal integration sites and potentially underwent multiple integration events, which are subject to ‘position effects’ that result in highly variable gene expression across a transformant population [27,31,34]. Overall expression was therefore stated as the mean of 20 transformants per construct, pre-selected from the initial population for the most robust fluorescence of the YFP reporter on the agar plate level. We confirmed here that expression is enhanced in a copy-number dependent manner for the LHCBM1i2 as was previously observed for the RBCS2i1 [31]. Expression was notably increased when 3 copies of a stimulating intron were inserted into the *Pc*Ps (vector D and E, Fig 5), demonstrating that the effect of IME is conserved across different introns and clearly additive. A correlation was observed between the intensity of IME and the promoter distance of intron insertions (Fig 4).

For repeated insertions, each intron even far downstream of the TSS contributed to enhanced expression (Vector D and E). It was previously discovered that RBCS2 intron 1 and the second intron (RBCS2i2) synergistically coordinate higher transgene expression than when i1 is used in the same frequency [31,33]. Here, we placed RBCS2i2 into the coding region of the C-terminal Strep-tag II to enable insertion of two more intron copies to be placed within the reporter (Fig 5). Additional intron insertions induced strong expression regardless of intron sequence used (vector F and G), however, this was not further distinguished between the respective increases derived from individual insertions. Strong fluorescence was clearly detectable already on the agar plate level for 14 and 16% of the initial transformants, respectively (Fig 5B). In the pooled high-expressing transformant population, up to 44-fold higher mRNA abundance was observed compared to the intronless *Pc*Ps construct. This increase is likely due to a combination of strategic intron insertions and reduced exon lengths which may be more in agreement with the host genome architecture than the other, although codon optimized, intronless CDSs [28,31]. We demonstrate that the bottleneck of limited nuclear transgene expression can be (partially) overcome by an intron-mediated increase in mRNA abundance (Fig 5). Increased target mRNA appears to be effectively translated to higher protein levels, which correlates to higher recombinant metabolite production levels for the *Pc*Ps. As the number and position of introns can correlate to predictable increases in transgene expression, the described gene design strategy could be used to fine-tune constitutive transgene expression as an alternative to employing promoters of varying strengths.

Although intron insertion had previously only been considered for RBCS2i1, IME has been clearly demonstrated also for the LHCBM1i2 in this study and likely works with any stimulating intron as shown in Fig 1. LHCBM1i2 exhibited stronger IME than RBCS2i1 in the smaller *sh*ble reporter, however, the overall *Pc*Ps expression yield between the two introns was comparable when exon lengths were minimized throughout the gene. It is unclear whether this is due to an upper limit of this specific target protein, or whether the mechanism of IME is simply fully activated.

## Materials and methods

### Selection of candidate introns sequences

Endogenous candidate introns were strategically selected from the 12 highest expressed genes under regular growth conditions (RBCS1, RBCS2, LHCBM1, LHCBM7, RPS8, PCY1, LHCBM3, RPP2, RPP1, RPL3, RPL10, RPL20) identified in a transcriptome study investigating the response in *C*. *reinhardtii* to nitrogen limitation [48]. If present, the first three intron sequences were selected per gene. Additionally, this set was completed with described *C*. *reinhardtii* introns (ACTIN [31], ARG7 [32], ßTUB2 [45] and RPL23 [80]) from current literature. Exogenous introns were identified from literature or established organism-specific expression vectors and were selected to represent stimulating introns from several evolutionary diverse organisms or their respective viruses, including mammals (Intron 1, 3, 4 and 12), plants (Intron 2, 10 and 11), humans (Intron 5, 8 and 13), fungi (Intron 6 and 7) and insects (Intron 9).

### PCR, cloning and vector design

*C*. *reinhardtii* endogenous introns were PCR amplified (Q5 High-Fidelity DNA Polymerase, NEB) from gDNA samples extracted via Chelex-100 method [81] from strain CC-124. Exogenous introns as well as modified intron sequences (e.g. internal deletions or splice site modifications), were assembled *de novo* using overlapping oligos. Resulting intron sequences contained respective overhangs (HindIII/SmaI) for subsequent cloning into the CDS of the bleomycin-resistance protein from *Streptoalloteichus hindustanus* (*sh*ble, NCBI: MG052655, [41]) located in the pOptimized vector system [41,46] carrying the following modifications: I) The intronless PSAD promoter [23] was used to drive expression (MluI/HindIII sites), II) The existing RBCS2i1 from the *sh*ble coding sequence was removed, and III) a previously designed N-terminal extension [31] was included, containing the start codon, a nucleotide coding region for an 8 amino acid long GS-linker and an additional SmaI site in frame with the *sh*ble CDS (Fig 1). For insertion of the RBCS2i1 and LHCBM1i2 into different insertion positions, the respective cloning sites (MluI, HindIII, SmaI, XhoI, HindIII) were used for integration (Fig 4).

Vectors A-G (Fig 5) were designed using the template vector pOpt2_mVenus_Paro [41,46] with following modifications: I) The HSP70/RBCS2 promoter was replaced by the intronless PSAD promoter located between XbaI/NdeI sites. II) The RBCS2i2 within the mVenus was removed and replaced by two copies of the RBCS2i1 or LHCBM1i2 (vector F and G, Fig 5). III) Both factor Xa-sites flanking the mVenus in the pOptimized vector were replaced by a 6 amino acid long GS-linker sequence. IV) The downstream-located Strep-tag II-epitope was extended by a 6 amino acid long GS-linker containing a copy of the RBCS2i2 (vector F and G, Fig 5, sequence in S3 Data). The scarless RBCS2i1 and LHCBM1i2 insertions into the coding sequence of the *Pogostemon cablin* Benth. patchoulol synthase (UniProt: Q49SP3, [31,79]) and of the mVenus reporter were performed using overlap extension (oe)PCR techniques [82] as previously described [31].

Cloning was performed using FastDigest restriction enzymes (Thermo Scientific), followed by product separation on 2% (w/v) agarose gels, DNA extraction (peqGOLD Gel Extraction Kit, VWR) and Ligation (Quick Ligation kit, M2200S, NEB) following manufacturer's instructions. All obtained plasmids were used for heat-shock transformation of chemically competent *Escherichia coli* DH5a cells with subsequent selection on 300 mg l^−1^ ampicillin containing LB-agar plates. *E*. *coli* colonies were checked by colony PCR and plasmid DNA was isolated from overnight cultures (peqGOLD Plasmid Miniprep Kit I, VWR). All sequences were confirmed by Sanger sequencing (Sequencing Core Facility, CeBiTec, Bielefeld University).

### *C*. *reinhardtii* cultivation and transformation

All *C*. *reinhardtii* cell lines were routinely maintained on TAP-agar plates [83] illuminated with 150 μmol photons m^−2^ s^−1^ light intensity. Liquid cultivations were performed in Erlenmeyer flasks on an orbital shaker using TAP medium and 250 μmol photons m^−2^ s^−1^ light intensity. *C*. *reinhardtii* UVM4 [24] was used for nuclear transformations via glass bead method [37].

Transformation efficiency experiments were performed in triplicates with 3 μg of linearized plasmid DNA and a recovery phase overnight at low light. Regenerated cells were transferred on TAP-agar plates containing 10 mg l^-1^ zeocin (Invivogen) and after 6 days obtained colonies were quantified using ImageJ software [84]. For comparison, absolute counts were normalized, relative to the respective intronless control transformation efficiency which was performed concurrent for each transformation experiment.

Transformations using vector A-G (Fig 5) were regenerated on TAP-agar plates containing 10 mg l^-1^ paromomycin (Sigma Aldrich). For each construct, 576 obtained colonies were transferred on fresh agar plates and screened in fluorescence measurements.

### Fluorescence measurements, flow cytometry and microscopy

Initial fluorescence detection was performed using the plant imaging system NightShade LB 985 (Berthold Technologies) with respective YFP-fluorescence filter sets (excitation: 504/10 nm, emission: 530/20 nm). Expression efficiency was grouped into three thresholds: low expression from 850–1500 relative fluorescent units (rfu), medium expression from 1500–3000 rfu, and high expression >3000 rfu.

From the initial transformant population, the 20 transformants with highest rfu for each construct were selected, individually cultivated in 24-well microtiter plates until logarithmic phase was reached, and quantitative construct expression intensities were determined based on fluorescence signals from a plate reader normalized to the respective cell densities. Flow cytometry analysis was performed as previously described [31].

### RNA extraction and RTqPCR

Isolated transformants were individually cultivated in 6-well microtiter plates until mid-logarithmic phase. Equal amounts were harvested by centrifugation (3000 xg, 3 min) and total RNA was extracted via phenol chloroform method followed by ethanol precipitation. For each sample, 3 μg total RNA were subjected to DNaseI digests (RQ1 RNase-Free DNase (Promega) and qPCR (Hi-ROX SensiFAST SYBR One-Step Kit, Bioline) or cDNA synthesis (BioScript Reverse Transcriptase, Bioline) prior to PCR amplifications. Relative mRNA amounts were quantified with target-specific oligos: mVenus: for 5′-TGCAGGAGCGCACCATCT-3′ and reverse 5′-GGCCCAGGATGTTGCCGTC-3′; 18S: for 5′-ACCTGGTTGATCCTGCCAG-3′ and reverse 5′-TGATCCTTCCGCAGGTTCAC-3′, [85]. SYBR Green fluorescence was recorded by a StepOnePlus Real-Time PCR System (Thermo Scientific) and relative mRNA expression levels were determined according to the 2^–ΔΔCt^ method [86]. Mean abundance was determined from technical triplicates of pooled RNA samples.

### SDS-PAGE and Western Blotting

For protein analysis, isolated transformants were cultivated in 6-well microtiter plates until mid-logarithmic growth phase, 4x10^7^ cells were harvested by centrifugation (3000 xg, 3 min) and pellets were resuspended in 200 μL 2xSDS sample buffer (60 mM Tris pH 6.8, 4% (w/v) SDS, 20% (v/v) glycerol, 0.01% (w/v) bromophenol blue). Equal protein amounts were separated in 10% polyacrylamide gels during Tris-glycine-SDS-PAGE [87]. Gels were stained using Colloidal Coomassie Brilliant Blue G-250 [88] or were subjected to Western blotting (semi-dry method, Trans-Blot Biorad, blotting buffer: 25 mM Tris, 192 mM glycine, 20% methanol) on 0.45 μm Protran Nitrocellulose membranes (Amersham). Overnight blocking was performed using blocking buffer (5% (w/v) BSA and 5% (w/v) milk powder in TBS) followed by immunodetection via Strep-tag II monoclonal antibody (α-Strep II, Iba Life Science 2-1509-001; 1:5.000 in blocking solution). Sequential washing steps were performed (TBS with 0.1% Tween 20) prior to addition of Pierce ECL Western Blotting substrate (Thermo Scientific) and detection in a Fusion FX Imaging system (Thermo Scientific).

### Two phase cultivations and product quantification

Transformants were individually cultivated for 6 days in constant light in 50 mL TAP medium supplemented with 5% (v/v) dodecane (Sigma Aldrich) as an organic solvent overlay. Dodecane fractions were harvested by centrifugation and analysed in GC/MS as previously described [40].

## Supporting information

S1 Fig Intron retention study and TSS definition. (A) Amplification scheme of 3 primer pairs (Position 1–3) at the indicated construct positions. (B) Total RNA samples were used for PCR amplification after DNaseI digest and reverse transcription. A corresponding sample (-RT) without addition of reverse transcriptase was included as a control. PCR products and RNA samples were separated on a 2% agarose gel under non-denatured conditions. Shown is the exemplary result for amplification of position 2. (C) PCR products after amplification of different 5‘UTR lengths (Position 1–3). Amplification was performed with cDNA samples from parental strain and representative transformants for intron RBCS2i1 (145 bp), RBCS2i2 (329 bp), LHCBM1i2 (253 bp) and ßTUB2i3 (137 bp) as well as corresponding intron-containing plasmid DNA. The expected size of processed cDNA and intronless DNA is indicated on the right. M– 1 kbp plus ladder (NEB)
(PDF)

S2 Fig Relative transformation efficiency of four selected introns (RBCS2i1 RBCS2i2, LHCBM1i2 and ßTUB2i3) with different IME capacities. Selection after transformation was performed at different light intensities (150, 350 and 700 μmol photons m^-2^ s^-1^). Data represents the mean of biological triplicates.
(PDF)

S3 Fig Relative transformation efficiency after addition of RBCS2i1_E2. (A) Relative transformation efficiency of RBCS2i1 after deletion of the E2 sequence element (RBCS2i1ΔE2) and after addition of one or two copies of the E2 (RBCS2i1, RBCS2i1_2xE2, RBCS2i1_3xE2) compared to the unchanged RBCS2i1 and the intronless control. (B) Relative transformation efficiency of the exogenous intron 12 (synthetic murine intron) and after addition of RBCS2i1E2.
(PDF)

S4 Fig Multiple Sequence Alignment of the six endogenous introns with the highest IME found in this work. (PDF)

S5 Fig Motif based sequence analysis via Multiple Em for Motif Elicitation tool (MEME, version 5.1.1) performed with six endogenous introns exhibiting the highest IME found in this work. (PDF)

S6 Fig Motif based sequence analysis via Multiple Em for Motif Elicitation tool (MEME, version 5.1.1) performed with 16 endogenous introns exhibiting an IME of 2 or higher compared to the intronless control found in this work. (PDF)

S7 Fig Relative transformation efficiency of 33 endogenous introns from highly expressed genes in *C*. *reinhardtii* compared to the intronless control in gene-clustered format. (PDF)

S1 Data FASTA format sequence information for endogenous and non-native introns used in this study. (DOCX)

S2 Data FASTA format sequence information of the modified *sh*ble screening vector. (DOCX)

S3 Data FASTA format sequence information of modified pOptimized vector F and G (Fig 5) carrying a full intron-containing *Pc*Ps. (DOCX)
