# Supplementary material for: Introns mediate post-transcriptional enhancement of nuclear gene expression in the green microalga Chlamydomonas reinhardtii
Source: PLoS Genet. 2020 Jul 30;16(7):e1008944. doi: 10.1371/journal.pgen.1008944 (PMC7419008; doi:10.1371/journal.pgen.1008944)

S1 Fig: Intron retention study and TSS definition. (A) Amplification scheme of 3 primer pairs (Position 1-3) at the indicated construct positions. (B) Total RNA samples were used for PCR amplification after DNaseI digest and reverse transcription. A corresponding sample (-RT) without addition of reverse transcriptase was included as a control. PCR products and RNA samples were separated on a 2% agarose gel under non-denatured conditions. Shown is the exemplary result for amplification of position 2. (C) PCR products after amplification of different 5'UTR lengths (Position 1-3). Amplification was performed with cDNA samples from parental strain and representative transformants for intron RBCS2i1 (145 bp), RBCS2i2 (329 bp), LHCBM1i2 (253 bp) and ßTUB2i3 (137 bp) as well as corresponding intron-containing plasmid DNA. The expected size of processed cDNA and intronless DNA is indicated on the right. M – 1 kbp plus ladder (NEB)

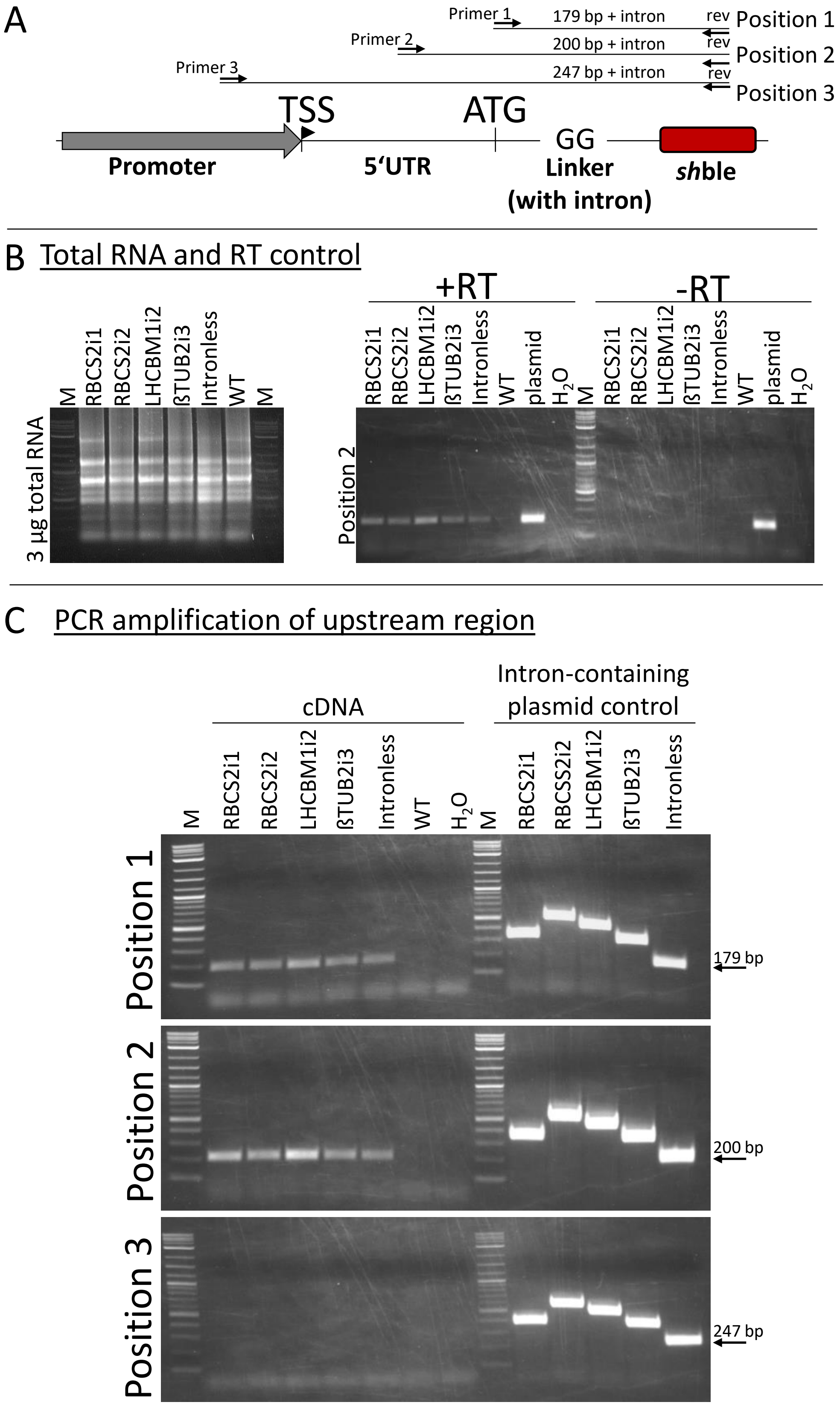

Supplement: S1 Fig — (A) Amplification scheme of 3 primer pairs (Position 1–3) at the indicated construct positions. (B) Total RNA samples were used for PCR amplification after DNaseI digest and reverse transcription. A corresponding sample (-RT) without addition of reverse transcriptase was included as a control. PCR products and RNA samples were separated on a 2% agarose gel under non-denatured conditions. Shown is the exemplary result for amplification of position 2. (C) PCR products after amplification of different 5‘UTR lengths (Position 1–3). Amplification was performed with cDNA samples from parental strain and representative transformants for intron RBCS2i1 (145 bp), RBCS2i2 (329 bp), LHCBM1i2 (253 bp) and ßTUB2i3 (137 bp) as well as corresponding intron-containing plasmid DNA. The expected size of processed cDNA and intronless DNA is indicated on the right. M– 1 kbp plus ladder (NEB) (PDF) [file pgen.1008944.s001.pdf]
