# Supplementary material for: Introns mediate post-transcriptional enhancement of nuclear gene expression in the green microalga Chlamydomonas reinhardtii
Source: PLoS Genet. 2020 Jul 30;16(7):e1008944. doi: 10.1371/journal.pgen.1008944 (PMC7419008; doi:10.1371/journal.pgen.1008944)

S2 Fig: Relative transformation efficiency of four selected introns (RBCS2i1 RBCS2i2, LHCBM1i2 and ßTUB2i3) with different IME capacities. Selection after transformantion was performed at different light intensities (150, 350 and 700 µmol photons m^{-2} s^{-1}). Data represents the mean of biological triplicates.

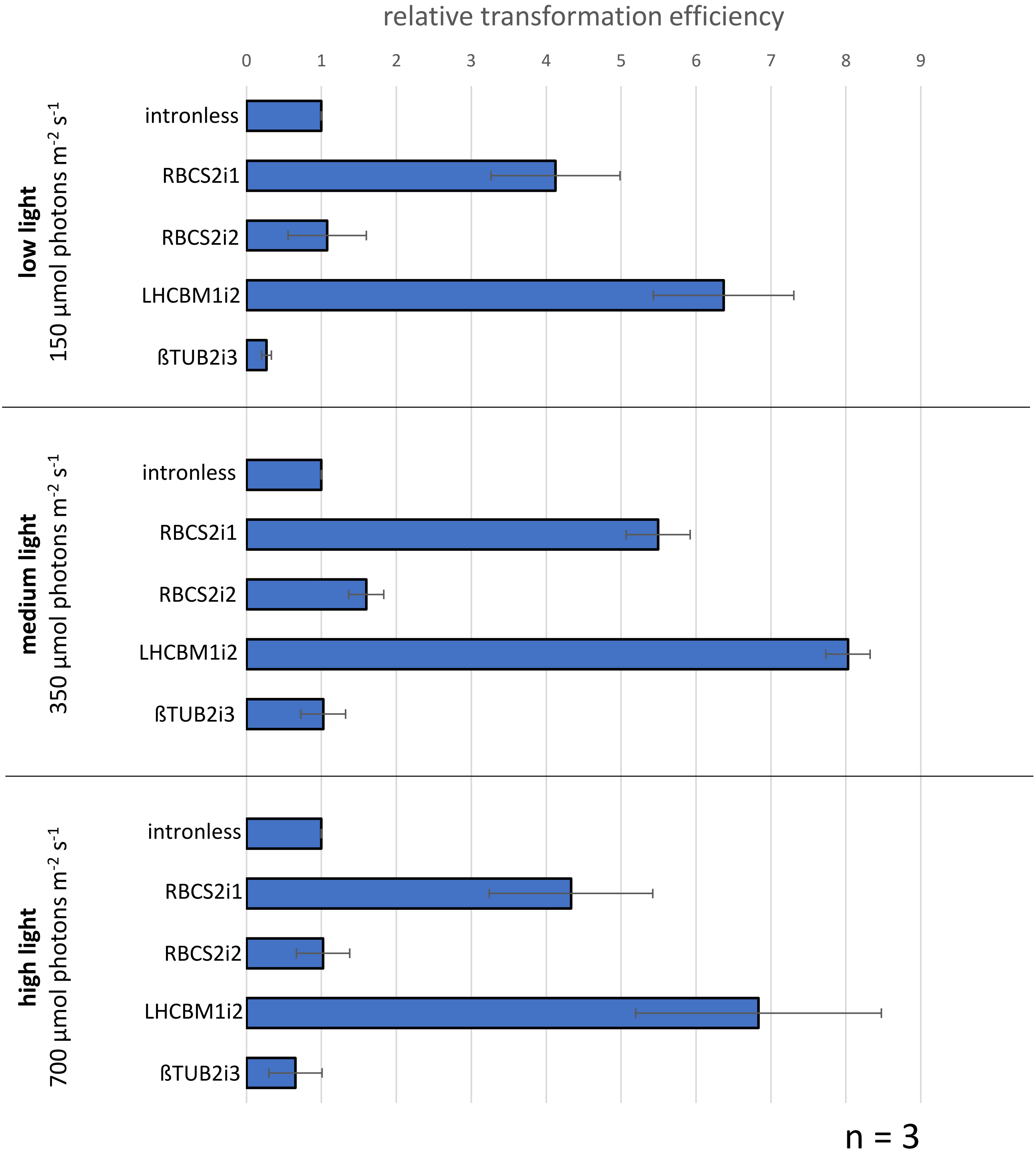

Supplement: S2 Fig — Selection after transformation was performed at different light intensities (150, 350 and 700 μmol photons m-2 s-1). Data represents the mean of biological triplicates. (PDF) [file pgen.1008944.s002.pdf]
