# Supplementary material for: Introns mediate post-transcriptional enhancement of nuclear gene expression in the green microalga Chlamydomonas reinhardtii
Source: PLoS Genet. 2020 Jul 30;16(7):e1008944. doi: 10.1371/journal.pgen.1008944 (PMC7419008; doi:10.1371/journal.pgen.1008944)

S3 Fig: Relative transformation efficiency after addition of RBCS2i1_E2. (A) Relative transformantion efficency of RBCS2i1 after deletion of the E2 sequence element (RBCS2i1ΔE2) and after addition of one or two copies of the E2 (RBCS2i1, RBCS2i1_2xE2, RBCS2i1_3xE2) compared to the unchanged RBCS2i1 and the intronless control. (B) Relative transformantion efficency of the exogenous intron 12 (synthetic murine intron) and after addition of RBCS2i1E2.

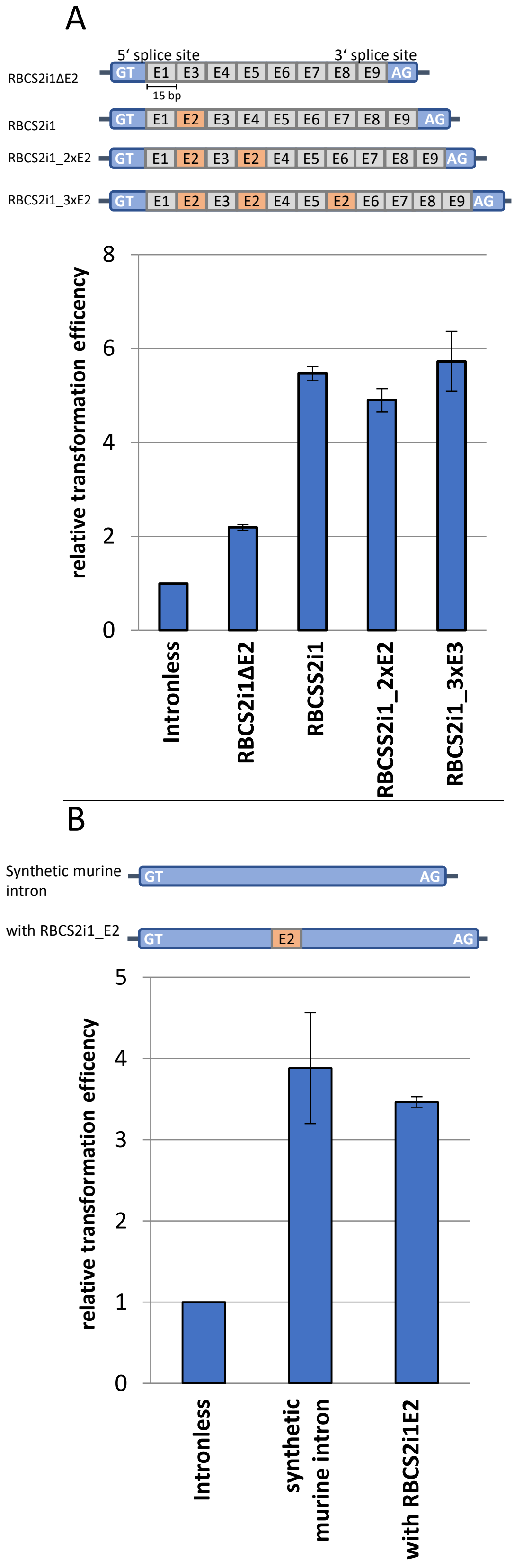

Supplement: S3 Fig — (A) Relative transformation efficiency of RBCS2i1 after deletion of the E2 sequence element (RBCS2i1ΔE2) and after addition of one or two copies of the E2 (RBCS2i1, RBCS2i1_2xE2, RBCS2i1_3xE2) compared to the unchanged RBCS2i1 and the intronless control. (B) Relative transformation efficiency of the exogenous intron 12 (synthetic murine intron) and after addition of RBCS2i1E2. (PDF) [file pgen.1008944.s003.pdf]
