# Supplementary material for: Introns mediate post-transcriptional enhancement of nuclear gene expression in the green microalga Chlamydomonas reinhardtii
Source: PLoS Genet. 2020 Jul 30;16(7):e1008944. doi: 10.1371/journal.pgen.1008944 (PMC7419008; doi:10.1371/journal.pgen.1008944)
Supplement: S4 Fig — (PDF) [file pgen.1008944.s004.pdf]

S4 Fig: Multiple Sequence Alignment of the six endogenous introns with the highest IME from the analysed data set.

```
CLUSTAL O(1.2.4) multiple sequence alignment


LHCBM1i2      -------------------GTAAGTCTTTCTGTGTCGCGGGGTTCTGGGCGT--TCGCAT      39
RPL10i1       GTGAGCTCTTTTTCTGAGTAGAAACTTTCCTTTCTGGGCTTGCTTCTTCGGCGACTTGAC      60
RPL3i1        -------------GTGAGTAGAATCTGTTACCTTTCATAGAGTTACCCCACA--AGCGCC      45
LHCBM7i1      -------------------------------------GTGAGTCGCTCCCGA--CGCGCA      21
RBCS1i1       -------------------------------------GTGAGTCGACGAGTA--AGCGCA      21
RBCS2i1       -------------------------------------GTGAGTCGACGAGCA--AGCCCG      21
                                                       *

LHCBM1i2      GCGCAACAGTGTCGCACGGTCGCTCTTGCAGCACAGTCACTACAGATAGTCCAAGTCCGA      99
RPL10i1       GCGTCTAGCTTAGCTCGCTCGATTTTCGCTTCTACGTAGTGTTAAC--------------      106
RPL3i1        GCACGAGAGTGCGCGGCGGCCGGACTCGCCA------AACAAAAGAGCTTAAACGGGCTG      99
LHCBM7i1      TGCGCGGACTCGCTGCTCCTCTCTCTGCCTT------GTGTCCAGATCCTTGCATGCATC      75
RBCS1i1       GCCCGAAGGATAGGGATTCTGCAAGTCGCGA------CGCA------------------A      57
RBCS2i1       GCGGATCAGGCAGCGTGCTTGCAGATTTGAC------TTGCAACGC----------CCGC      65
                                       *

LHCBM1i2      CGCATGGCGATCGGGCAACTGCGATTTGCACATGCGGCAAGGGATCTCTAGCTCGGGCTG      159
RPL10i1       ----------------GATAGCTTATGAAGCAAGTTGACAATTAAGGCACAGGGCAGGAG      150
RPL3i1        AGCATTTTCACGCAGTTTATTCTATATGATCGCTGC---TAGTAGATGGCACCGCCGATA      156
LHCBM7i1      AG---GCACATGGAGCTCACTGC-----------TT---TGGTACATGCTATTGGCCTGG      118
RBCS1i1       TTGCTTGGGGCCCAGCCTG----------------------CTGCCTCACATCGC-----      90
RBCS2i1       ATTGTGTCGACGAAGGCTT----------------------TTGGCTCCTCTGTC-----      98


LHCBM1i2      GCGAAGCCT------------TCAGGACATGGAGCGCTGTCCAGCAGCTGGTTGGTGATG      207
RPL10i1       TCGCCGCCGGCACAAGTCGCGCCGGGTTTTACGCTCGTCGCA--------CGCGCTGCTG      202
RPL3i1        TGGAAGCCGACAGGCGCAGCGTCTGTGGGATCCTTTTCTTGACGCTCTTGGACCTTGATG      216
LHCBM7i1      TCTATGCGCTTCAGATTAGCTGCCTTCACCTGCTTTCGTTGG------------------      160
RBCS1i1       ------ACGTGCTCTGCCACTTCTAACGAGTACTGGTCAATC------------------      126
RBCS2i1       -----GCTGTCTCAAGCAGCATCTAACCCTGCGTCGCCGTTT------------------      135
                                    *

LHCBM1i2      CTCTATCCTAAATTGCCCCTCCCACACACCCTTACTTGCTTTCCAG-   253
RPL10i1       ACGCTCTGTAATTTTATGTTGCCTCCCGT---TTATTGCAG------   240
RPL3i1        AAGTGTGCTAACTTCAATTTCGTGGCTGCCTGTGATTTCGTTTGCAG   263
LHCBM7i1      ---TCTCCATCCTGACCTTGTCGCGTCCAACAAATTTTGCCTTGCAG   204
RBCS1i1       GCGTGATCGCAG-----------------------------------   138
RBCS2i1       CCATTTGCAG-------------------------------------   145
```
