# Supplementary material for: Introns mediate post-transcriptional enhancement of nuclear gene expression in the green microalga Chlamydomonas reinhardtii
Source: PLoS Genet. 2020 Jul 30;16(7):e1008944. doi: 10.1371/journal.pgen.1008944 (PMC7419008; doi:10.1371/journal.pgen.1008944)

S5 Fig: Motif based sequence analysis via Multiple Em for Motif Elicitation tool (MEME, version 5.1.1) performed with six endogenous introns exhibiting the highest IME from the analysed data set.

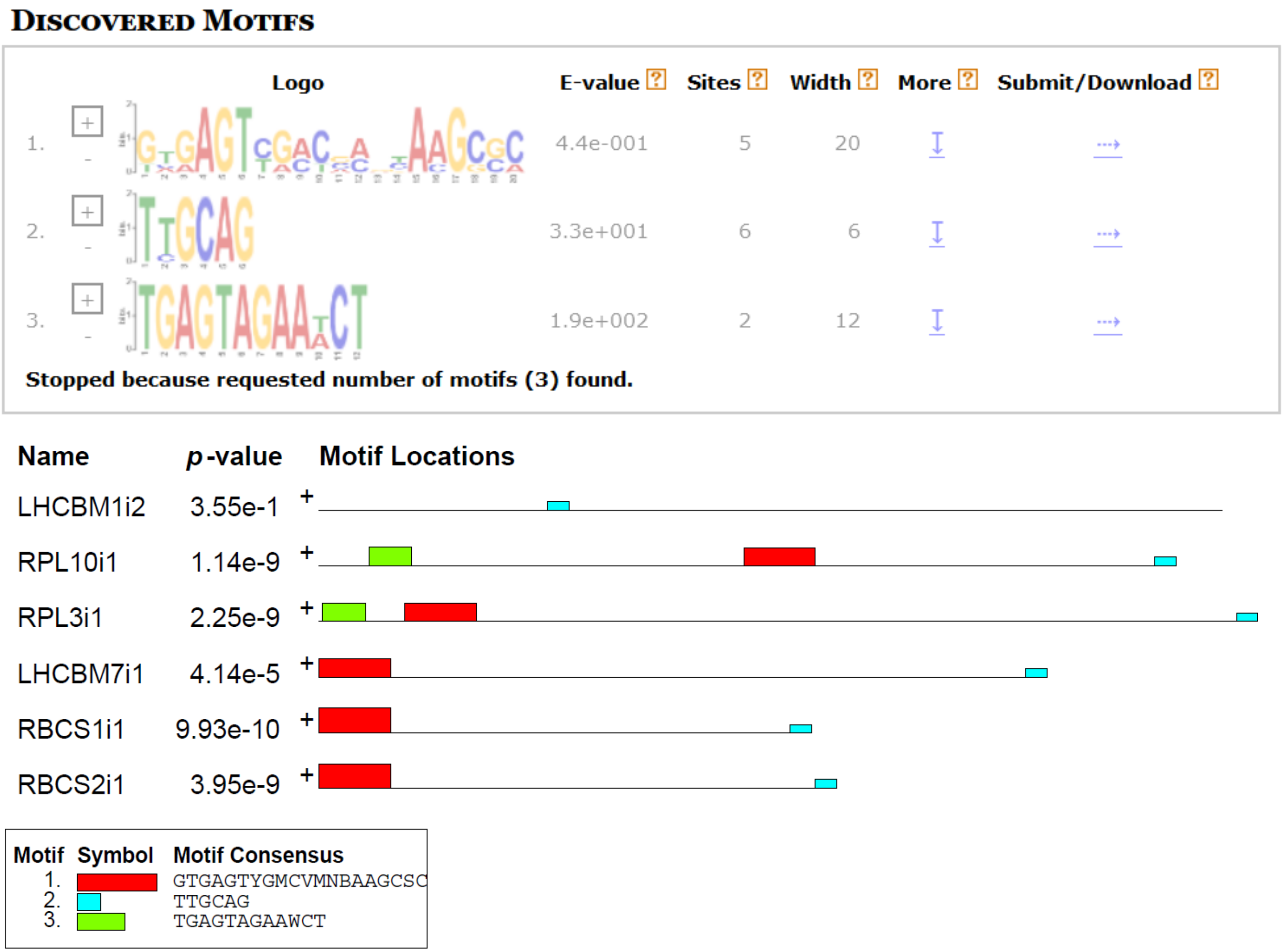

Supplement: S5 Fig — (PDF) [file pgen.1008944.s005.pdf]
