# Supplementary material for: Introns mediate post-transcriptional enhancement of nuclear gene expression in the green microalga Chlamydomonas reinhardtii
Source: PLoS Genet. 2020 Jul 30;16(7):e1008944. doi: 10.1371/journal.pgen.1008944 (PMC7419008; doi:10.1371/journal.pgen.1008944)

S7 Fig: Relative transformation efficiency of 33 endogenous introns from highly expressed genes in *C. reinhardtii* compared to the intronless control.

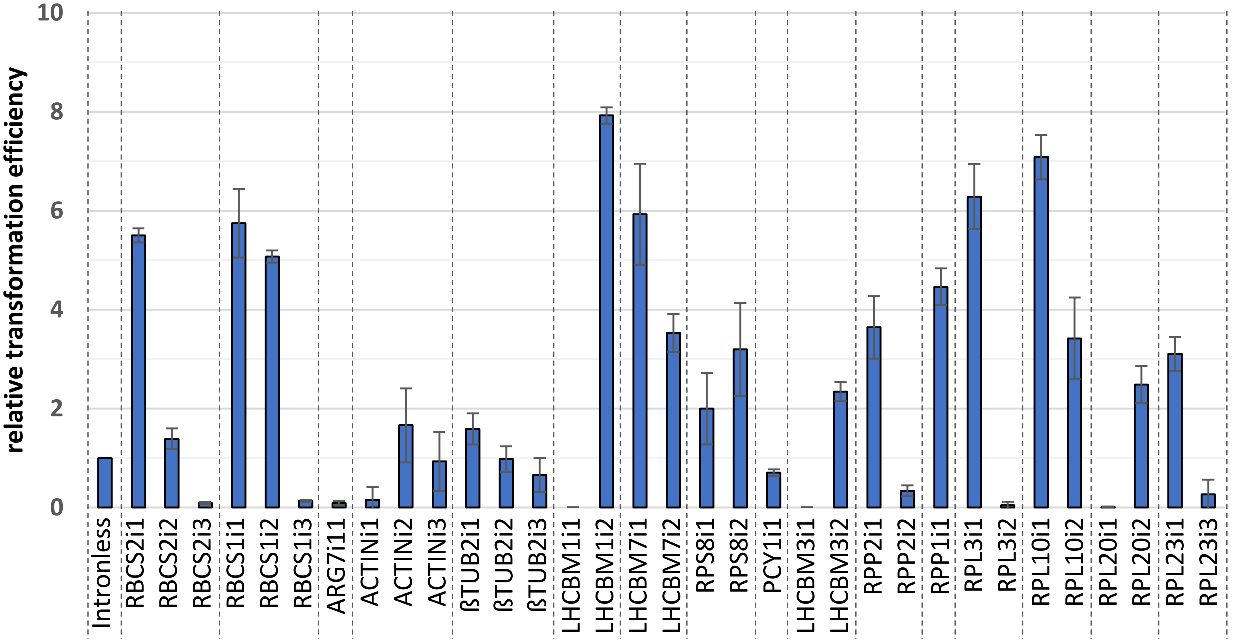

Supplement: S7 Fig — (PDF) [file pgen.1008944.s007.pdf]
