# Supplementary material for: Introns mediate post-transcriptional enhancement of nuclear gene expression in the green microalga Chlamydomonas reinhardtii
Source: PLoS Genet. 2020 Jul 30;16(7):e1008944. doi: 10.1371/journal.pgen.1008944 (PMC7419008; doi:10.1371/journal.pgen.1008944)
Supplement: S1 Data — (DOCX) [file pgen.1008944.s008.docx]

S1 Data. FASTA format sequence information for endogenous and non-native introns used in this study.

***C. reinhardtii* endogenous intron sequences**

>RBCS2i1_Cre02.g120150.t1.2

Gtgagtcgacgagcaagcccggcggatcaggcagcgtgcttgcagatttgacttgcaacgcccgcattgtgtcgacgaaggcttttggctcctctgtcgctgtctcaagcagcatctaaccctgcgtcgccgtttccatttgcag

>RBCS2i2_Cre02.g120150.t1.2

Gtgagcttgcggggttgcgagcaacactccagcaacgaacagtgcccaagtcaggaatctgcagtcagcctgggctttcggcggctttttcttgggcaaacagcttgcactcatgccagcgcggcttgtccagcctcacttgagctttccagctgctaccagccgggctatacgacagcgacagagccatagcgtggaatcacttatttgggttgccgaagtagcggtcggagcgtgagttcttggtcaagccgccccttatccggttcctgtccgtgtctttgtccctcgttcacccttcgcggcacccttcatccccttgcttgcag

>RBCS2i3_Cre02.g120150.t1.2

Gtaagtctggcgagagcccgacgggtccactgtggcactgggttagcttttggcacacgggtccactgtggcactggttagcttggcaccgggacagcgcctatctcaccgcggggaactgacgcatacccctgctcgtgcttcagcacggaaaagcaaggggcccaattccatctttggtggttctgtgcgctggtgactgaacctcttctccctcccatttcccgtgcgcccgcag

>RBCS1i1_Cre02.g120100.t1.2

Gtgagtcgacgagtaagcgcagcccgaaggatagggattctgcaagtcgcgacgcaattgcttggggcccagcctgctgcctcacatcgcacgtgctctgccacttctaacgagtactggtcaatcgcgtgatcgcag

>RBCS1i2_Cre02.g120100.t1.2

Gtgagcacggcttggtgccgtgggcatatgtagctgggctgtgtttgagagctagttaatacgatctagggcgtagctgcagactgcgacattacgatcgctcgaagcttgagattagcttgttcgttgtctgctgtatagtttgcctgtcatccgcccttatctgggatagccctggactgcttgcgtcggccacttatcttacccccttcgtgctgtcgcttgcag

>RBCS1i3_Cre02.g120100.t1.2

gtaagtcggagcgagagccagaCGGGTCCACTGTGGCACTGGGTTAGCTTTTGGCACAcgggtccactgtggcactggttagcttggcaccgggacagcgcctatctcaccgcggggaactgacgcatacccctgctcgtgcttcagcacggaaaagcaaggggcccaattccatctttggtggttctgtgcgctggtgactgaacctcttctccctcccatttcccgtgcgcccgcag

>ARG7i11_Cre01.g021251.t1.1

Gtgcggccgggcgggagggcgtgagggcgtgggtggggcatgcccggggttgtgagagctatcgaacgttgtgccgcgcctgtttcacaatgtcgggccacagggtatgcagtttcctctccatatgtataacaaactgaccaccaatcatgcacgctcacacgctctcccacacacacgcgcaccacgccaccacag

>ACTINi1_Cre13.g603700.t1.2

gtagagagccaaacacctgcgcgtgcgttgcggtagagcctcgctgcgttgcctcgcttctgttgcagcatcgtagcacatcacattgaatggaaagggtagagttgtcatgcactagcgcaggcgaagaatgcttagctttgccgaggtggcagccctccgatcacttcgttctCgctctcgcggacgtgacacgcag

>ACTINi2_Cre13.g603700.t1.2

gtgagcaggtgttcagggcgcgtcgcCgcgacgaagcggAaaggtggccggactccttcgctggaaggtttggccgtcttgggcgcgccagcagctcaacttcgatctggtcattctaatgagcacaatcacacgtagtggcgtggggacttccatttaacttgggcgttgggcctggcgtgggccccgcgctgcagcgcgttgttcgagcaaacctgatgctggcgatcgctgtgtcgccgtgtccgcag

>ACTINi3_Cre13.g603700.t1.2

Gtgcgctcgcttttcgtttggctcgtgcatgcttgcacgcgttatatgcatgtatgaagccatgttgagcatacttgcttctttggactctgcag

>ßTUB2i1_Cre12.g549550.t1.2

gtgcgttgaagcgcttagcgcattggctgagggctagcgcagtcaaggggcgcggggtcgCggctacacccccgcggctcaatttcaaacctgtttccgacttcgaggctcatcgtcgctccgcctgcttgcgcctttacatccacag

>ßTUB2i2_Cre12.g549550.t1.2

gtgagttgcctgaagagttcctgttggggctgtagcgagccgcgggcaggttgctagcgctgatgctactgtGgtgctagcgcacgacttgtgttccttggagcgcgcgtttCtcggcaccgccgGttcccagccaatgcttgataatgtttttgccgtcatggtttggtgcttttgctcTgcctgatgtctgctcctcttcctCgctcggacatgcgggcgaatgcctgaccttcttcccttcccttttgctcctcag

>ßTUB2i3_Cre12.g549550.t1.2

gtgagcagttctgcacgggccgtcgggtgtcagtgtatggaccgcagggctTggcatgacgggctgctttgggctgtgcgccccttcgtcttcacacgttgctgacttctctctcccctgttcttccctaccctcag

>LHCBM1i1_Cre01.g066917.t1.1

Gtgtgtagctcgccaatgaagtgctctggggaagatttgcagggggaatgacagggcacgactgccggaactggccactccggcatggggatcctctttccctgacttgcgcttcctgtctggtgagctgccaccagtggcaaagaatacagctccatgtctacaatgcggcctgccgatttcactgtatcaagctcttgacgtctgaaccctttcgcgatggccctttgcttgcag

>LHCBM1i2_Cre01.g066917.t1.1

Gtaagtctttctgtgtcgcggggttctgggcgttcgcatgcgcaacagtgtcgcacggtcgctcttgcagcacagtcactacagatagtccaagtccgacgcatggcgatcgggcaactgcgatttgcacatgcggcaagggatctctagctcgggctggcgaagccttcaggacatggagcgctgtccagcagctggttggtgatgctctatcctaaattgcccctcccacacacccttacttgctttccag

>LHCBM7i1_Cre12.g548950.t1.2

gtgagtcgctcccgacgcgcatgcgcggactcgctgctcctctctctgccttgtgtccagatccttgcAtgcatcaggcacatggagctcactgctttggtacatgctattggcctggtctatgcgcttcagattagctgccttcacctgctttcgttggtcTccatcctgaccttgtcgcgtccaacaaattttgccttgcag

>LHCBM7i2_Cre12.g548950.t1.2

gtgagttacgcaagatggctcttcaAggaccgccttacgccttgcagctgagagcttcactgtgccttcaacctgacctgcaaaGgcttattacattcataagcaacgccagcagtgcggatctctggccatccttgctcggatatgcgaactcgatactgactttacctttgtcatgtcgtttgcttgcag

>RPS8i1_Cre06.g272800.t1.2

Gtgagtacatggggcaacttacagggctcttccgctcgcataatctagtcggctgccccggcatgcacttcgcagcggtctgttctggcccctgtcgtcgccatagtattgacagctcattccaaccattgttgcag

>RPS8i2_Cre06.g272800.t1.2

Gtgagctcgggccgtgttcgtagcacatcaggcgtgaagtattcaatattcagccttgggaagcagcaggcgtggggccgccgcagcgggccagccgagggaccatggcggctgtcgcgcagaccatgtaccgggaagcggaggcggctaagctaccagcaaatgcaaattttctgttttagaagtcatccgtgtgagcgatgtgcggtcggcgcactgggcagtgcagtcggtgggggcggcttcccttttgcccgggacaaagatggcgccactcgagttccgtgctgaaaacgcatgcgggtgattgctctgcag

>PCY1i1_Cre03.g182551.t1.2

Gtgagtctatcatgggttcatggtttcaattggccgggcagatcacgcagtttggttggctccggatgtgcgaatcgcatttgcttgcacagcgacataacgccgacagctcgcgcgaattcgtgtttgctgtgcacattgcgctagatttgcttgatcagtcgtaggggactcccaatccaccgtctatcgttagcggttcagcggggtgccacgtcctcagcgacatgcgacacgtccactgccgcgactgactttcctctccctcccctctctatctatgtgcag

>LHCBM3i1_Cre04.g232104.t1.1

Gtgtgcaagctagggtcgcacatcgcttagcgaaacaactcacgtcacctttgttgggctcatgggccctcgtggagcttcgggagctaacctgctggcgatggcgtgtctatgtgcag

>LHCBM3i2_Cre04.g232104.t1.1

Gtgagtgttccggcatgcgcggggcacgggttcgcgctgggcttcagtctcatggatccccttgcgggcttgggctcagtggcagtgttctagtcagcagcgccgcggggattcttgagttattgccaccgtttgcgcctgtggcttcgcacatggcaacccgcttaagcaacccttcaacgccttatgcggatcttcacttgctggactctaggcatgttctaggtacatcatatatgccatgcattgactaccataactaaatgcgcgccctccttcctctttgcttgctcttcag

>RPP2i1_Cre02.g143050.t1.2

Gtaagttatctgcttcgctcgcgcatcatttgccgtcggctcagcgtccgcacctctgaccgcag

>RPP2i2_Cre02.g143050.t1.2

Gtaagcagcgagccggcggcttgcctatggcggctcccgctctgtcggcggagcagctctctcaccgcgcggttgttgccacttgcag

>RPP1i1_Cre17.g738300.t1.2

Gtgcgtcgcgctcgcttacgtcgctaagcagcttgtatcttgtacattctatagctgacggtcgccgttttcgtttgcag

>RPL3i1_Cre10.g417700.t1.2

Gtgagtagaatctgttacctttcatagagttaccccacaagcgccgcacgagagtgcgcggcggccggactcgccaaacaaaagagcttaaacgggctgagcattttcacgcagtttattctatatgatcgctgctagtagatggcaccgccgatatggaagccgacaggcgcagcgtctgtgggatccttttcttgacgctcttggaccttgatgaagtgtgctaacttcaatttcgtggctgcctgtgatttcgtttgcag

>RPL3i2_Cre10.g417700.t1.2

Gtgcgtaaagcgcgctgttccagcgccgactgtgggtgggcgatgtgtgtcgctttgtgttgtgcgtggcgcactcggctgatatcgaatggtgacgctgaaggaaaacgtttaggaggaatgggtccggcatatccgcaaaagcggaacaagtcgacgcaaccttcttgttaagcggtatcacttggccgggcacgcag

>RPL10i1_Cre02.g101350.t1.2

Gtgagctctttttctgagtagaaactttcctttctgggcttgcttcttcggcgacttgacgcgtctagcttagctcgctcgattttcgcttctacgtagtgttaacgatagcttatgaagcaagttgacaattaaggcacagggcaggagtcgccgccggcacaagtcgcgccgggttttacgctcgtcgcacgcgctgctgacgctctgtaattttatgttgcctcccgtttattgcag

>RPL10i2_Cre02.g101350.t1.2

gtgagttggggcatcCttgagggaggcgcgaaagtggcggccgagaatggctgggttgccaggcggttccgtgcgacatactcaccgcaacattgcttttaataatacggtggtatcagattttgatgagcgcgcggattgccagacgtgagctaacccgaccccgacggccttcctcgccccacag

>RPL20i1_Cre16.g660150.t1.2

Gtgcgcgagcgggagcgatttcgccgccgacgctcgaaaagcagctgctttgcgctgcctctttgccttgcaatatgcatttcttatattcttaaagccaacattgtgtgcttgcgtcgcgcgcag

>RPL20i2_Cre16.g660150.t1.2

Gtaagcggtctgcaaaagccgccgccgacagtcaactgatgtcatgctcgctagtgacacctgcttaacaatgtattatttttcttcatcgcag

>RPL23i1_Cre04.g211800.t1.2

Gtaggcaaattgcattttcttttattcatttaggtccttgagaactccgctcacagctttctcttgtctgcgcgcag

>RPL23i3_Cre04.g211800.t1.2

gtgaggcgggggttcggagggactaggggaagagttggacgccagagtggggcgggttttcgggcctctgacatgcgcgcgtgccttcccaagcacaactcaccatctgactcgcatgctgacggtcctctgtgtcccttggcccttgcgcgcag

# Exogenous intron sequences

# >exogenous_intron_1_Ricinus_communis_catalase_intron 1

GTAAATTTCTAGTTTTTCTCCTTCATTTTCTTGGTTAGGACCCTTTTCTCTTTTTATTTTTTTGAGCTTTGATCTTTCTTTAAACTGATCTATTTTTTAATTGATTGGTTATGGTGTAAATATTACATAGCTTTAACTGATAATCTGATTACTTTATTTCGTGTGTCTATGATGATGATGATAGTTACAG

>exogenous_intron_2_human_adeno_virus_with_3‘_splice_site_from_human_Ig

gtgagtactccctctcaaaagcgggcatgacttctgcgctaagattgtcagtttccaaaaacgaggaggatttgatattcacctggcccgcggtgatgcctttgagggtggccgcgtccatctggtcagaaaagacaatctttttgttgtcaggcttgaggtgtggcaggcttgagGtctggccatacacttgagtgacaatgacatccactttgcctttctctccacag

>exogenous_intron_3_ human_Elongation_factor_1-alpha_1

TCGAGCTTTTGGAGTACGTCGTCTTTAGGTTGGGGGGAGGGGTTTTATGCGATGGAGTTTCCCCACACTGAGTGGGTGGAGACTGAAGTTAGGCCAGCTTGGCACTTGATGTAATTCTCCTTGGAATTTGCCCTTTTTGAGTTTGGATCTTGGTTCATTCTCAAGCCTCAGACAGTGGTTCAAAGTTTTTTTCTTCCATTTCAG

>exogenous_intron_4_ *A._nidulans_*glyceraldehyde-3-phosphate_dehydrogenase

GTAAGTACTTTGCTACATCCATACTCCATCCTTCCCATCCCTTATTCCTTTGAACCTTTCAGTTCGAGCTTTCCCACTTCATCGCAGCTTGACTAACAGCTACCCCGCTTGAGCAG

>exogenous_intron_5_ synthetic:_chicken_β-actin_(CBA)_and_mice_minute_virus

GGAGTCGCTGCGCGCTGCCTTCGCCCCGTGCCCCGCTCCGCCGCCGCCTCGCGCCGCCCGCCCCGGCTCTGACTGACCGCGTTACTCCCACAGGTGAGCGGGCGGGACGGCCCTTCTCCTCCGGGCTGTAATTAGCTGAGCAAGAGGTAAGGGTTTAAGGGATGGTTGGTTGGTGGGGTATTAATGTTTAATTACCTGGAGCACCTGCCTGAAATCACTTTTTTTCAG

>exogenous_intron_6_ Drosophila_myosin_heavy_chain_intron_16

GTAGGTTCAACCACTGATGCCTAGGCACACCGAAACGACTAACCCTAATTCTTATCCTTTACTTCAG

> exogenous_intron_7_Simian_Virus_40_intron

GTAAGTTTAGTCTTTTTGTCTTTTATTTCAGGTCCCGGTTCCGGTGGTGGTGCAAATCAAAGAACTGCTCCTCAGTGGATGTTGCCTTTACTTCTAG

> exogenous_intron_8_ human_cytomegalovirus_intron_A

GTAAGTACCGCCTATAGAGTCTATAGGCCCACCCCCTTGGCTTCTTATGCATGCTATACTGTTTTTGGCTTGGGGTCTATACACCCCCGCTTCCTCATGTTATAGGTGATGGTATAGCTTAGCCTATAGGTGTGGGTTATTGACCATTATTGACCACTCCAACGGTGGAGGGCAGTGTAGTCTGAGCAGTACTCGTTGCTGCCGCGCGCGCCACCAGACATAATAGCTGACAGACTAACAGACTGTTCCTTTCCATGGGTCTTTTCTGCAG

>exogenous_intron_9_SV40_small_T_antigen_intron

GTAAATATAAAATTTTTAAGTGTATAATGTGTTAAACTACTGATTCTAATTGTTTGTGTATTTTAG

>exogenous_intron_10_ *Zea_mays* alcohol_dehydrogenase_1

gtatctaatcagccatcccatttgtgatctttgtcagtagatatgatacaacaactcgcggttgacttgcgccttcttggcggcttatctgtcttag

>exogenous_intron_11_synthetic:_murine_concensus_intron

gtaagtcgactcgttggatcccagcgatatcaaaactagtgtgacgctgctgacccctttctttcccttctgcag

>exogenous_intron_12_ *A._thaliana_*ubiquitin_10_intron_1

GTAAATTTCTGTGTTCCTTATTCTCTCAAAATCTTCGATTTTGTTTTCGTTCGATCCCAATTTCGTATATGTTCTTTGGTTTAGATTCTGTTAATCTTAGATCGAAGACGATTTTCTGGGTTTGATCGTTAGATATCATCTTAATTCTCGATTAGGGTTTCATAGATATCATCCGATTTGTTCAAATAATTTGAGTTTTGTCGAATAATTACTCTTCGATTTGTGATTTCTATCTAGATCTGGTGTTAGTTTCTAGTTTGTGCGATCGAATTTGTCGATTAATCTGAGTTTTTCTGATTAACAG

>exogenous_intron_13_ *A._thaliana_*Cor15a_intron_1

GTAAGTCTACATTCTTTCTTCTTTTAGTATCTTGCCTCATAAGTAAGGATCTTAGCAGGCAATGTTTATGGTATACTATATTAGTATAGATTTTAGTGGAAATATGTTTGTTTTGAACTTATTTTATGATCATATTTGACTATTATCAAAGATAAAGATTCATATACCGTACATTATATATCTCTATTTTTCTAGTTTACATGTATAGCTCTAAGTTTATTTGATGATTCTGTTGACTACTTTTGGATATGTGTTTTGAAACCTTTGATAAATACTAAAATAAATTTTAATTTGAAAATGATATAG
