# Supplementary material for: Introns mediate post-transcriptional enhancement of nuclear gene expression in the green microalga Chlamydomonas reinhardtii
Source: PLoS Genet. 2020 Jul 30;16(7):e1008944. doi: 10.1371/journal.pgen.1008944 (PMC7419008; doi:10.1371/journal.pgen.1008944)
Supplement: S2 Data — (DOCX) [file pgen.1008944.s009.docx]

S2 Data. FASTA format sequence information of the modified *sh*ble screening vector.

LOCUS Exported 6333 bp ds-DNA circular SYN

DEFINITION synthetic circular DNA

ACCESSION .

VERSION .

KEYWORDS .

SOURCE synthetic DNA construct

ORGANISM synthetic DNA construct

REFERENCE 1 (bases 1 to 6333)

AUTHORS Thomas Baier

FEATURES Location/Qualifiers

source 1..6333

/organism="synthetic DNA construct"

/mol_type="other DNA"

source 2593..3355

/organism="synthetic DNA construct"

/mol_type="other DNA"

promoter 572..838

/label=HSP70Ap promoter

/label=HSP70Ap

promoter 845..1065

/label=RBCS2p promoter

/label=RBCS2p

transit_peptide join(1066..1073,1219)

/label=RBCS2ORF

intron 1074..1218

/label=rbcS2i1

CDS 1247..1258

/codon_start=1

/product="Factor Xa recognition and cleavage site"

/label=Factor Xa site

/translation="IEGR"

CDS join(1259..1562,1892..2295)

/codon_start=1

/label=mRuby2

/translation="VSKGEELIKENMRMKVVMEGSVNGHQFKCTGEGEGNPYMGTQTMRIKVIEGGPLPFAFDILATSFMYGSRTFIKYPKGIPDFFKQSFPEGFTWERVTRYEDGGVVTVMQDTSLEDGCLVYHVQVRGVNFPSNGPVMQKKTKGWEPNTEMMYPADGGLRGYTHMALKVDGGGHLSCSFVTTYRSKKTVGNIKMPGIHAVDHRLERLEESDNEMFVVQREHAVAKFAGLGGGMDELYK"

intron 1563..1891

/label=rbcS2i2

CDS 2296..2307

/codon_start=1

/product="Factor Xa recognition and cleavage site"

/label=Factor Xa site

/translation="IEGR"

CDS 2320..2343

/codon_start=1

/product="peptide that binds Strep-Tactin(R), an engineered form of streptavidin"

/label=Strep-Tag II

/translation="WSHPQFEK"

misc_feature 2347..2580

/label=3'UTR

promoter 2593..3355

/label=PSAD promoter

CDS 3389..3766

/codon_start=1

/label=ble

/translation="AKLTSAVPVLTARDVAGAVEFWTDRLGFSRDFVEDDFAGVVRDDVTLFISAVQDQDQVVPDNTLAWVWVRGLDELYAEWSEVVSTNFRDASGPAMTEIGEQPWG

REFALRDPAGNCVHFVAEEQD"

misc_feature 3776..4014

/label=3'UTR

/label=3'UTR(1)

rep_origin complement(4474..5062)

/direction=LEFT

/label=ori

/note="high-copy-number ColE1/pMB1/pBR322/pUC origin of

replication"

CDS complement(5233..6093)

/codon_start=1

/gene="bla"

/product="beta-lactamase"

/label=AmpR

/note="confers resistance to ampicillin, carbenicillin, and related antibiotics"

/translation="MSIQHFRVALIPFFAAFCLPVFAHPETLVKVKDAEDQLGARVGYIELDLNSGKILESFRPEERFPMMSTFKVLLCGAVLSRIDAGQEQLGRRIHYSQNDLVEYSPVTEKHLTDGMTVRELCSAAITMSDNTAANLLLTTIGGPKELTAFLHNMGDHVTRLDRWEPELNEAIPNDERDTTMPVAMATTLRKLLTGELLTLASRQQLIDWMEADKVAGPLLRSA

LPAGWFIADKSGAGERGSRGIIAALGPDGKPSRIVVIYTTGSQATMDERNRQIAEIGASLIKHW"

promoter complement(6094..6198)

/gene="bla"

/label=AmpR promoter

rep_origin complement(join(6225..6333,1..347))

/direction=LEFT

/label=f1 ori

/note="f1 bacteriophage origin of replication; arrow

indicates direction of (+) strand synthesis"

ORIGIN

1 atagaccgag atagggttga gtgttgttcc agtttggaac aagagtccac tattaaagaa

61 cgtggactcc aacgtcaaag ggcgaaaaac cgtctatcag ggcgatggcc cactacgtga

121 accatcaccc taatcaagtt ttttggggtc gaggtgccgt aaagcactaa atcggaaccc

181 taaagggagc ccccgattta gagcttgacg gggaaagccg gcgaacgtgg cgagaaagga

241 agggaagaaa gcgaaaggag cgggcgctag ggcgctggca agtgtagcgg tcacgctgcg

301 cgtaaccacc acacccgccg cgcttaatgc gccgctacag ggcgcgtccc attcgccatt

361 caggctgcgc aactgttggg aagggcgatc ggtgcgggcc tcttcgctat tacgccagct

421 ggcgaaaggg ggatgtgctg caaggcgatt aagttgggta acgccagggt tttcccagtc

481 acgacgttgt aaaacgacgg ccagtgagcg cgcgtaatac gactcactat agggcgaatt

541 ggagctccac cgcggtggcg gccgctctag agctgaggct tgacatgatt ggtgcgtatg

601 tttgtatgaa gctacaggac tgatttggcg ggctatgagg gcgggggaag ctctggaagg

661 gccgcgatgg ggcgcgcggc gtccagaagg cgccatacgg cccgctggcg gcacccatcc

721 ggtataaaag cccgcgaccc cgaacggtga cctccacttt cagcgacaaa cgagcactta

781 tacatacgcg actattctgc cgctatacat aaccactcag ctagcttaag atcccatcac

841 cggtgcatgc cgggcgcgcc agaaggagcg cagccaaacc aggatgatgt ttgatggggt

901 atttgagcac ttgcaaccct tatccggaag ccccctggcc cacaaaggct aggcgccaat

961 gcaagcagtt cgcatgcagc ccctggagcg gtgccctcct gataaaccgg ccagggggcc

1021 tatgttcttt acttttttac aagagaagtc actcaacatc ttaaaatggc caggtgagtc

1081 gacgagcaag cccggcggat caggcagcgt gcttgcagat ttgacttgca acgcccgcat

1141 tgtgtcgacg aaggcttttg gctcctctgt cgctgtctca agcagcatct aaccctgcgt

1201 cgccgtttcc atttgcagga tgcatatggg atccagatct gacgtcatcg agggcagggt

1261 gagcaagggc gaggagctga tcaaggagaa catgcgcatg aaggtggtga tggagggcag

1321 cgtgaacggc caccagttca agtgcaccgg cgagggcgag ggcaacccct acatgggcac

1381 ccagaccatg cgcatcaagg tgatcgaggg cggccccctg cccttcgcct tcgacatcct

1441 ggccaccagc ttcatgtacg gcagccgcac cttcatcaag taccccaagg gcatccccga

1501 cttcttcaag cagagcttcc ccgagggctt cacctgggag cgcgtgaccc ggtacgagga

1561 cggtgagctt gcggggttgc gagcaacact ccagcaacga acagtgccca agtcaggaat

1621 ctgcagtcag cctgggcttt cggcggcttt ttcttgggca aacagcttgc actcatgcca

1681 gcgcggcttg tccagcctca cttgagcttt ccagctgcta ccagccgggc tatacgacag

1741 cgacagagcc atagcgtgga atcacttatt tgggttgccg aagtagcggt cggagcgtga

1801 gttcttggtc aagccgcccc ttatccggtt cctgtccgtg tctttgtccc tcgttcaccc

1861 ttcgcggcac ccttcatccc cttgcttgca ggtggcgtgg tgaccgtgat gcaggacacc

1921 agcctggagg acggctgcct ggtgtaccac gtgcaggtgc gcggcgtgaa cttccccagc

1981 aacggccccg tgatgcagaa gaagaccaag ggctgggagc ccaacaccga gatgatgtac

2041 cccgccgacg gcggcctgcg cggctacacc cacatggccc tgaaggtgga cggcggcggc

2101 cacctgagct gcagcttcgt gaccacctac cgcagcaaga agaccgtggg caacatcaag

2161 atgcccggca tccacgccgt ggaccaccgc ctggagcgcc tggaggagag cgacaacgag

2221 atgttcgtgg tgcagcgcga gcacgccgtg gccaagttcg ccggcctggg cggcggcatg

2281 gacgagctgt acaagatcga gggcagggat atcgaattct ggagccaccc gcagttcgag

2341 aagtaaccgc tccgtgtaaa tggaggcgct cgttgatctg agccttgccc cctgacgaac

2401 ggcggtggat ggaagatact gctctcaagt gctgaagcgg tagcttagct ccccgtttcg

2461 tgctgatcag tctttttcaa cacgtaaaaa gcggaggagt tttgcaattt tgttggttgt

2521 aacgatcctc cgttgatttt ggcctctttc tccatgggcg ggctgggcgt atttgaagcg

2581 actagtacgc gtaccaatcg tcacacgagc cctcgtcaga aacacgtctc cgccacgctc

2641 tccctctcac ggccgacccc gcagcccttt tgccctttcc taggccaccg acaggaccca

2701 ggcgctctca gcatgcctca acaacccgta ctcgtgccag cggtgccctt gtgctggtga

2761 tcgcttggaa gcgcatgcga agacgaaggg gcggagcagg cggcctggct gttcgaaggg

2821 ctcgccgcca gttcgggtgc ctttctccac gcgcgcctcc acacctaccg atgcgtgaag

2881 gcaggcaaat gctcatgttt gcccgaactc ggagtcctta aaaagccgct tcttgtcgtc

2941 gttccgagac atgttagcag atcgcagtgc cacctttcct gacgcgctcg gccccatatt

3001 cggacgcaat tgtcatttgt agcacaattg gagcaaatct ggcgaggcag taggctttta

3061 agttgcaagg cgagagagca aagtgggacg cggcgtgatt attggtattt acgcgacggc

3121 ccggcgcgtt agcggccctt cccccaggcc agggacgatt atgtatcaat attgttgcgt

3181 tcgggcactc gtgcgagggc tcctgcgggc tggggagggg gatctgggaa ttggaggtac

3241 gaccgagatg gcttgctcgg ggggaggttt cctcgccgag caagccaggg ttaggtgttg

3301 cgctcttgac tcgttgtgca ttctaggacc ccactgctac tcacaacaag cccataagct

3361 tatgggcagc ggcggcagcg gccccggggc caagctgacc agcgccgttc cggtgctcac

3421 cgcgcgcgac gtggccggag cggtcgagtt ctggaccgac cggctcgggt tcagccggga

3481 cttcgtggag gacgacttcg ccggtgtggt ccgggacgac gtgaccctgt tcatcagcgc

3541 ggtccaggac caggaccagg tggtgccgga caacaccctg gcctgggtgt gggtgcgcgg

3601 cctggacgag ctgtacgccg agtggtcgga ggtcgtgtcc acgaacttcc gggacgcctc

3661 cgggccggcc atgaccgaga tcggcgagca gccgtggggg cgggagttcg ccctgcgcga

3721 cccggccggc aactgcgtgc acttcgtggc cgaggagcag gactaactcg agtgaccgct

3781 ccgtgtaaat ggaggcgctc gttgatctga gccttgcccc ctgacgaacg gcggtggatg

3841 gaagatactg ctctcaagtg ctgaagcggt agcttagctc cccgtttcgt gctgatcagt

3901 ctttttcaac acgtaaaaag cggaggagtt ttgcaatttt gttggttgta acgatcctcc

3961 gttgattttg gcctctttct ccatgggcgg gctgggcgta tttgaagcgg acccggtacc

4021 cagcttttgt tccctttagt gagggttaat tgcgcgcttg gcgtaatcat ggtcatagct

4081 gtttcctgtg tgaaattgtt atccgctcac aattccacac aacatacgag ccggaagcat

4141 aaagtgtaaa gcctggggtg cctaatgagt gagctaactc acattaattg cgttgcgctc

4201 actgcccgct ttccagtcgg gaaacctgtc gtgccagctg cattaatgaa tcggccaacg

4261 cgcggggaga ggcggtttgc gtattgggcg ctcttccgct tcctcgctca ctgactcgct

4321 gcgctcggtc gttcggctgc ggcgagcggt atcagctcac tcaaaggcgg taatacggtt

4381 atccacagaa tcaggggata acgcaggaaa gaacatgtga gcaaaaggcc agcaaaaggc

4441 caggaaccgt aaaaaggccg cgttgctggc gtttttccat aggctccgcc cccctgacga

4501 gcatcacaaa aatcgacgct caagtcagag gtggcgaaac ccgacaggac tataaagata

4561 ccaggcgttt ccccctggaa gctccctcgt gcgctctcct gttccgaccc tgccgcttac

4621 cggatacctg tccgcctttc tcccttcggg aagcgtggcg ctttctcata gctcacgctg

4681 taggtatctc agttcggtgt aggtcgttcg ctccaagctg ggctgtgtgc acgaaccccc

4741 cgttcagccc gaccgctgcg ccttatccgg taactatcgt cttgagtcca acccggtaag

4801 acacgactta tcgccactgg cagcagccac tggtaacagg attagcagag cgaggtatgt

4861 aggcggtgct acagagttct tgaagtggtg gcctaactac ggctacacta gaaggacagt

4921 atttggtatc tgcgctctgc tgaagccagt taccttcgga aaaagagttg gtagctcttg

4981 atccggcaaa caaaccaccg ctggtagcgg tggttttttt gtttgcaagc agcagattac

5041 gcgcagaaaa aaaggatctc aagaagatcc tttgatcttt tctacggggt ctgacgctca

5101 gtggaacgaa aactcacgtt aagggatttt ggtcatgaga ttatcaaaaa ggatcttcac

5161 ctagatcctt ttaaattaaa aatgaagttt taaatcaatc taaagtatat atgagtaaac

5221 ttggtctgac agttaccaat gcttaatcag tgaggcacct atctcagcga tctgtctatt

5281 tcgttcatcc atagttgcct gactccccgt cgtgtagata actacgatac gggagggctt

5341 accatctggc cccagtgctg caatgatacc gcgagaccca cgctcaccgg ctccagattt

5401 atcagcaata aaccagccag ccggaagggc cgagcgcaga agtggtcctg caactttatc

5461 cgcctccatc cagtctatta attgttgccg ggaagctaga gtaagtagtt cgccagttaa

5521 tagtttgcgc aacgttgttg ccattgctac aggcatcgtg gtgtcacgct cgtcgtttgg

5581 tatggcttca ttcagctccg gttcccaacg atcaaggcga gttacatgat cccccatgtt

5641 gtgcaaaaaa gcggttagct ccttcggtcc tccgatcgtt gtcagaagta agttggccgc

5701 agtgttatca ctcatggtta tggcagcact gcataattct cttactgtca tgccatccgt

5761 aagatgcttt tctgtgactg gtgagtactc aaccaagtca ttctgagaat agtgtatgcg

5821 gcgaccgagt tgctcttgcc cggcgtcaat acgggataat accgcgccac atagcagaac

5881 tttaaaagtg ctcatcattg gaaaacgttc ttcggggcga aaactctcaa ggatcttacc

5941 gctgttgaga tccagttcga tgtaacccac tcgtgcaccc aactgatctt cagcatcttt

6001 tactttcacc agcgtttctg ggtgagcaaa aacaggaagg caaaatgccg caaaaaaggg

6061 aataagggcg acacggaaat gttgaatact catactcttc ctttttcaat attattgaag

6121 catttatcag ggttattgtc tcatgagcgg atacatattt gaatgtattt agaaaaataa

6181 acaaataggg gttccgcgca catttccccg aaaagtgcca cactaaattg taagcgttaa

6241 tattttgtta aaattcgcgt taaatttttg ttaaatcagc tcatttttta accaataggc

6301 cgaaatcggc aaaatccctt ataaatcaaa aga

//
