# Supplementary material for: Introns mediate post-transcriptional enhancement of nuclear gene expression in the green microalga Chlamydomonas reinhardtii
Source: PLoS Genet. 2020 Jul 30;16(7):e1008944. doi: 10.1371/journal.pgen.1008944 (PMC7419008; doi:10.1371/journal.pgen.1008944)
Supplement: S3 Data — (DOCX) [file pgen.1008944.s010.docx]

S3 Data. FASTA format sequence information of modified pOptimized vector F and G (Figure 5) carrying a full intron-containing *Pc*Ps.

Vector sequence F (Figure 5)

LOCUS Exported 9730 bp ds-DNA circular SYN

DEFINITION synthetic circular DNA

ACCESSION .

VERSION .

KEYWORDS .

SOURCE synthetic DNA construct

ORGANISM synthetic DNA construct

REFERENCE 1 (bases 1 to 9730)

AUTHORS Thomas Baier

FEATURES Location/Qualifiers

source 1..9730

/organism="synthetic DNA construct"

/mol_type="other DNA"

misc_feature 572..1334

/label=PSAD promoter

gene join(1347..1784,2038..2538,2792..3265,3519..3767)

/label=Patchoulol synthase

misc_feature 1785..2037

/label=LHCBM1i2

misc_feature 2539..2791

/label=LHCBM1i2

misc_feature 3266..3518

/label=LHCBM1i2

CDS 3780..3797

/label=GSGSGS-Linker

gene join(3798..3996,4250..4566,4820..5017)

/label=mVenus

misc_feature 3997..4249

/label=LHCBM1i2

misc_feature 4567..4819

/label=LHCBM1i2

CDS 5018..5035

/codon_start=1

/label=GSGSGS-Linker

/translation="GSGSGS"

CDS join(5048..5066,5396..5397)

/codon_start=1

/label=GSGS-Linker

/translation="GSGSGSG"

intron 5067..5395

/label=RBCS2 intron 2

CDS 5398..5421

/codon_start=1

/product="peptide that binds Strep-Tactin(R), an engineered form of streptavidin"

/label=Strep-Tag II

/translation="WSHPQFEK"

misc_feature 5425..5658

/label=3'UTR

misc_feature 5666..5670

/label=MCS

promoter 5671..5937

/label=HSP70Ap

/label=HSP70Ap(1)

promoter join(5944..6172,6318..6319)

/label=RBCS2p promoter

/label=RBCS2p

intron 6173..6317

/label=RBCS2 intron 1

CDS 6360..7163

/codon_start=1

/label=APHVIII

/translation="MDDALRALRGRYPGCEWVVVEDGASGAGVYRLRGGGRELFVKVAALGAGVGLLGEAERLVWLAEVGIPVPRVVEGGGDERVAWLVTEAVPGRPASARWPREQRLDVAVALAGLARSLHALDWERCPFDRSLAVTVPQAARAVAEGSVDLEDLDEERKGWSGERLLAELERTRPADEDLAVCHGDLCPDNVLLDPRTCEVTGLIDVGRVGRADRHSDLALVLR

ELAHEEDPWFGPECSAAFLREYGRGWDGAVSEEKLAFYRLLDEFF"

misc_feature 7173..7411

/label=3'UTR

/label=3'UTR(1)

rep_origin complement(7871..8459)

/direction=LEFT

/label=ori

/note="high-copy-number ColE1/pMB1/pBR322/pUC origin of

replication"

CDS complement(8630..9490)

/codon_start=1

/gene="bla"

/product="beta-lactamase"

/label=AmpR

/note="confers resistance to ampicillin, carbenicillin, and related antibiotics"

/translation="MSIQHFRVALIPFFAAFCLPVFAHPETLVKVKDAEDQLGARVGYIELDLNSGKILESFRPEERFPMMSTFKVLLCGAVLSRIDAGQEQLGRRIHYSQNDLVEYSPVTEKHLTDGMTVRELCSAAITMSDNTAANLLLTTIGGPKELTAFLHNMGDHVTRLDRWEPELNEAIPNDERDTTMPVAMATTLRKLLTGELLTLASRQQLIDWMEADKVAGPLLRSALPAGWFIADKSGAGERGSRGIIAALGPDGKPSRIVVIYTTGSQATMDERNRQIAEIGASLIKHW"

promoter complement(9491..9595)

/gene="bla"

/label=AmpR promoter

rep_origin complement(join(9622..9730,1..347))

/direction=LEFT

/label=f1 ori

/note="f1 bacteriophage origin of replication; arrow

indicates direction of (+) strand synthesis"

ORIGIN

1 atagaccgag atagggttga gtgttgttcc agtttggaac aagagtccac tattaaagaa

61 cgtggactcc aacgtcaaag ggcgaaaaac cgtctatcag ggcgatggcc cactacgtga

121 accatcaccc taatcaagtt ttttggggtc gaggtgccgt aaagcactaa atcggaaccc

181 taaagggagc ccccgattta gagcttgacg gggaaagccg gcgaacgtgg cgagaaagga

241 agggaagaaa gcgaaaggag cgggcgctag ggcgctggca agtgtagcgg tcacgctgcg

301 cgtaaccacc acacccgccg cgcttaatgc gccgctacag ggcgcgtccc attcgccatt

361 caggctgcgc aactgttggg aagggcgatc ggtgcgggcc tcttcgctat tacgccagct

421 ggcgaaaggg ggatgtgctg caaggcgatt aagttgggta acgccagggt tttcccagtc

481 acgacgttgt aaaacgacgg ccagtgagcg cgcgtaatac gactcactat agggcgaatt

541 ggagctccac cgcggtggcg gccgctctag aaccaatcgt cacacgagcc ctcgtcagaa

601 acacgtctcc gccacgctct ccctctcacg gccgaccccg cagccctttt gccctttcct

661 aggccaccga caggacccag gcgctctcag catgcctcaa caacccgtac tcgtgccagc

721 ggtgcccttg tgctggtgat cgcttggaag cgcatgcgaa gacgaagggg cggagcaggc

781 ggcctggctg ttcgaagggc tcgccgccag ttcgggtgcc tttctccacg cgcgcctcca

841 cacctaccga tgcgtgaagg caggcaaatg ctcatgtttg cccgaactcg gagtccttaa

901 aaagccgctt cttgtcgtcg ttccgagaca tgttagcaga tcgcagtgcc acctttcctg

961 acgcgctcgg ccccatattc ggacgcaatt gtcatttgta gcacaattgg agcaaatctg

1021 gcgaggcagt aggcttttaa gttgcaaggc gagagagcaa agtgggacgc ggcgtgatta

1081 ttggtattta cgcgacggcc cggcgcgtta gcggcccttc ccccaggcca gggacgatta

1141 tgtatcaata ttgttgcgtt cgggcactcg tgcgagggct cctgcgggct ggggaggggg

1201 atctgggaat tggaggtacg accgagatgg cttgctcggg gggaggtttc ctcgccgagc

1261 aagccagggt taggtgttgc gctcttgact cgttgtgcat tctaggaccc cactgctact

1321 cacaacaagc ccatcatatg ggatccgagc tgtacgccca gagcgtgggc gtgggcgccg

1381 ccagccgccc cctggccaac ttccacccct gcgtgtgggg cgacaagttc atcgtgtaca

1441 acccccagag ctgccaggcc ggcgagcgcg aggaggccga ggagctgaag gtggagctga

1501 agcgcgagct gaaggaggcc agcgacaact acatgcgcca gctgaagatg gtggacgcca

1561 tccagcgcct gggcatcgac tacctgttcg tggaggacgt ggacgaggcc ctgaagaacc

1621 tgttcgagat gttcgacgcc ttctgcaaga acaaccacga catgcacgcc accgccctga

1681 gcttccgcct gctgcgccag cacggctacc gcgtgtcctg cgaggtgttc gagaagttca

1741 aggacggcaa ggacggcttc aaggtgccca acgaggacgg cgcggtaagt ctttctgtgt

1801 cgcggggttc tgggcgttcg catgcgcaac agtgtcgcac ggtcgctctt gcagcacagt

1861 cactacagat agtccaagtc cgacgcatgg cgatcgggca actgcgattt gcacatgcgg

1921 caagggatct ctagctcggg ctggcgaagc cttcaggaca tggagcgctg tccagcagct

1981 ggttggtgat gctctatcct aaattgcccc tcccacacac ccttacttgc tttccaggtg

2041 gcggtgctgg agttcttcga ggccacccac ctgcgcgtgc acggcgagga cgtgctggac

2101 aacgccttcg acttcacccg caactacctg gagagcgtgt acgccaccct gaacgacccc

2161 accgccaagc aggtgcacaa cgcgctgaac gagttctcct tccgccgcgg cctgccccgc

2221 gtggaggccc gcaagtacat cagcatctac gagcagtacg ccagccacca caagggcctg

2281 ctgaagctgg ccaagctgga cttcaacctg gtgcaggcgc tgcaccgccg cgagctgtcc

2341 gaggacagcc gctggtggaa gaccctgcag gtgcccacca agctgagctt cgtgcgcgac

2401 cgcctggtgg agagctactt ctgggccagc ggcagctact tcgagcccaa ctacagcgtg

2461 gcccgcatga tcctggcgaa gggcctggcc gtgctgagcc tgatggacga cgtgtacgac

2521 gcctacggca ccttcgaggt aagtctttct gtgtcgcggg gttctgggcg ttcgcatgcg

2581 caacagtgtc gcacggtcgc tcttgcagca cagtcactac agatagtcca agtccgacgc

2641 atggcgatcg ggcaactgcg atttgcacat gcggcaaggg atctctagct cgggctggcg

2701 aagccttcag gacatggagc gctgtccagc agctggttgg tgatgctcta tcctaaattg

2761 cccctcccac acacccttac ttgctttcca ggagctgcag atgttcaccg acgcgatcga

2821 gcgctgggac gccagctgcc tggacaagct gcccgactac atgaagatcg tgtacaaggc

2881 cctgctggac gtgttcgagg aggtggacga ggagctgatc aagctgggcg ccccctaccg

2941 cgcctactac ggcaaggagg ccatgaagta cgccgcccgc gcctacatgg aggaggccca

3001 gtggcgcgag cagaagcaca agcccaccac caaggagtac atgaagctgg cgaccaagac

3061 ctgcggctac atcaccctga tcatcctgtc ctgcctgggc gtggaggagg gcatcgtgac

3121 caaggaggcg ttcgactggg tgttcagccg cccgccgttc atcgaggcga ccctgatcat

3181 cgcccgcctg gtcaacgaca tcaccggcca cgagttcgag aagaagcgcg agcacgtgcg

3241 caccgccgtg gagtgctaca tggaggtaag tctttctgtg tcgcggggtt ctgggcgttc

3301 gcatgcgcaa cagtgtcgca cggtcgctct tgcagcacag tcactacaga tagtccaagt

3361 ccgacgcatg gcgatcgggc aactgcgatt tgcacatgcg gcaagggatc tctagctcgg

3421 gctggcgaag ccttcaggac atggagcgct gtccagcagc tggttggtga tgctctatcc

3481 taaattgccc ctcccacaca cccttacttg ctttccagga gcacaaggtg ggcaagcagg

3541 aggtggtgtc cgagttctac aaccagatgg agagcgcctg gaaggacatc aacgagggct

3601 tcctgcgccc cgtggagttc cccatccccc tgctgtacct gatcctgaac agcgtgcgca

3661 ccctggaggt gatctacaag gagggcgaca gctacaccca cgtgggcccg gccatgcaga

3721 acatcatcaa gcagctgtac ctgcaccccg tgccctacgg cagcggcaga tctgacgtcg

3781 gcagcggcag cggcagcgtg agcaagggcg aggagctgtt caccggcgtg gtgcccatcc

3841 tggtggagct ggacggcgac gtgaacggcc acaagttcag cgtgagcggc gagggcgagg

3901 gcgacgccac ctacggcaag ctgaccctga agctgatctg caccaccggc aagctgcccg

3961 tgccctggcc caccctggtg accaccctgg gctacggtaa gtctttctgt gtcgcggggt

4021 tctgggcgtt cgcatgcgca acagtgtcgc acggtcgctc ttgcagcaca gtcactacag

4081 atagtccaag tccgacgcat ggcgatcggg caactgcgat ttgcacatgc ggcaagggat

4141 ctctagctcg ggctggcgaa gccttcagga catggagcgc tgtccagcag ctggttggtg

4201 atgctctatc ctaaattgcc cctcccacac acccttactt gctttccagg cctgcagtgc

4261 ttcgcccgct accccgacca catgaagcag cacgacttct tcaagagcgc catgcccgag

4321 ggctacgtgc aggagcgcac catcttcttc aaggacgacg gtaactacaa gacccgcgcc

4381 gaggtgaagt tcgagggcga caccctggtg aaccgcatcg agctgaaggg catcgacttc

4441 aaggaggacg gcaacatcct gggccacaag ctggagtaca actacaacag ccacaacgtg

4501 tacatcaccg ccgacaagca gaagaacggc atcaaggcca acttcaagat ccgccacaac

4561 atcgaggtaa gtctttctgt gtcgcggggt tctgggcgtt cgcatgcgca acagtgtcgc

4621 acggtcgctc ttgcagcaca gtcactacag atagtccaag tccgacgcat ggcgatcggg

4681 caactgcgat ttgcacatgc ggcaagggat ctctagctcg ggctggcgaa gccttcagga

4741 catggagcgc tgtccagcag ctggttggtg atgctctatc ctaaattgcc cctcccacac

4801 acccttactt gctttccagg acggcggcgt gcagctggcc gaccactacc agcagaacac

4861 ccccatcggc gacggccccg tgctgctgcc cgacaaccac tacctgagct accagagcaa

4921 gctgagcaag gaccccaacg agaagcgcga ccacatggtg ctgctggagt tcgtgaccgc

4981 cgccggcatc accctgggca tggacgagct gtacaagggc agcggcagcg gcagcgatat

5041 cgaattcggc agcggcagcg gctcaggtga gcttgcgggg ttgcgagcaa cactccagca

5101 acgaacagtg cccaagtcag gaatctgcag tcagcctggg ctttcggcgg ctttttcttg

5161 ggcaaacagc ttgcactcat gccagcgcgg cttgtccagc ctcacttgag ctttccagct

5221 gctaccagcc gggctatacg acagcgacag agccatagcg tggaatcact tatttgggtt

5281 gccgaagtag cggtcggagc gtgagttctt ggtcaagccg ccccttatcc ggttcctgtc

5341 cgtgtctttg tccctcgttc acccttcgcg gcacccttca tccccttgct tgcaggttgg

5401 agccacccgc agttcgagaa gtaaccgctc cgtgtaaatg gaggcgctcg ttgatctgag

5461 ccttgccccc tgacgaacgg cggtggatgg aagatactgc tctcaagtgc tgaagcggta

5521 gcttagctcc ccgtttcgtg ctgatcagtc tttttcaaca cgtaaaaagc ggaggagttt

5581 tgcaattttg ttggttgtaa cgatcctccg ttgattttgg cctctttctc catgggcggg

5641 ctgggcgtat ttgaagcgac tagtacgcgt gctgaggctt gacatgattg gtgcgtatgt

5701 ttgtatgaag ctacaggact gatttggcgg gctatgaggg cgggggaagc tctggaaggg

5761 ccgcgatggg gcgcgcggcg tccagaaggc gccatacggc ccgctggcgg cacccatccg

5821 gtataaaagc ccgcgacccc gaacggtgac ctccactttc agcgacaaac gagcacttat

5881 acatacgcga ctattctgcc gctatacata accactcagc tagcttaaga tcccatccct

5941 agggcatgcc gggcgcgcca gaaggagcgc agccaaacca ggatgatgtt tgatggggta

6001 tttgagcact tgcaaccctt atccggaagc cccctggccc acaaaggcta ggcgccaatg

6061 caagcagttc gcatgcagcc cctggagcgg tgccctcctg ataaaccggc cagggggcct

6121 atgttcttta cttttttaca agagaagtca ctcaacatct taaaatggcc aggtgagtcg

6181 acgagcaagc ccggcggatc aggcagcgtg cttgcagatt tgacttgcaa cgcccgcatt

6241 gtgtcgacga aggcttttgg ctcctctgtc gctgtctcaa gcagcatcta accctgcgtc

6301 gccgtttcca tttgcaggaa gcttactccg ccctccccgg tgctgaagaa tttcgaagca

6361 tggacgatgc gttgcgtgca ctgcggggtc ggtatcccgg ttgtgagtgg gttgttgtgg

6421 aggatggggc ctcgggggct ggtgtttatc ggcttcgggg tggtgggcgg gagttgtttg

6481 tcaaggtggc agctctgggg gccggggtgg gcttgttggg tgaggctgag cggctggtgt

6541 ggttggcgga ggtggggatt cccgtacctc gtgttgtgga gggtggtggg gacgagaggg

6601 tcgcctggtt ggtcaccgaa gcggttccgg ggcgtccggc cagtgcgcgg tggccgcggg

6661 agcagcggct ggacgtggcg gtggcgctcg cggggctcgc tcgttcgctg cacgcgctgg

6721 actgggagcg gtgtccgttc gatcgcagtc tcgcggtgac ggtgccgcag gcggcccgtg

6781 ctgtcgctga agggagcgtc gacttggagg atctggacga ggagcggaag gggtggtcgg

6841 gggagcggct tctcgccgag ctggagcgga ctcggcctgc ggacgaggat ctggcggttt

6901 gccacggtga cctgtgcccg gacaacgtgc tgctcgaccc tcgtacctgc gaggtgaccg

6961 ggctgatcga cgtggggcgg gtcggccgtg cggaccggca ctccgatctc gcgctggtgc

7021 tgcgcgagct ggcccacgag gaggacccgt ggttcgggcc ggagtgttcc gcggcgttcc

7081 tgcgggagta cgggcgcggg tgggatgggg cggtatcgga ggaaaagctg gcgttttacc

7141 ggctgttgga cgagttcttc tgactcgagt gaccgctccg tgtaaatgga ggcgctcgtt

7201 gatctgagcc ttgccccctg acgaacggcg gtggatggaa gatactgctc tcaagtgctg

7261 aagcggtagc ttagctcccc gtttcgtgct gatcagtctt tttcaacacg taaaaagcgg

7321 aggagttttg caattttgtt ggttgtaacg atcctccgtt gattttggcc tctttctcca

7381 tgggcgggct gggcgtattt gaagcggacc cggtacccag cttttgttcc ctttagtgag

7441 ggttaattgc gcgcttggcg taatcatggt catagctgtt tcctgtgtga aattgttatc

7501 cgctcacaat tccacacaac atacgagccg gaagcataaa gtgtaaagcc tggggtgcct

7561 aatgagtgag ctaactcaca ttaattgcgt tgcgctcact gcccgctttc cagtcgggaa

7621 acctgtcgtg ccagctgcat taatgaatcg gccaacgcgc ggggagaggc ggtttgcgta

7681 ttgggcgctc ttccgcttcc tcgctcactg actcgctgcg ctcggtcgtt cggctgcggc

7741 gagcggtatc agctcactca aaggcggtaa tacggttatc cacagaatca ggggataacg

7801 caggaaagaa catgtgagca aaaggccagc aaaaggccag gaaccgtaaa aaggccgcgt

7861 tgctggcgtt tttccatagg ctccgccccc ctgacgagca tcacaaaaat cgacgctcaa

7921 gtcagaggtg gcgaaacccg acaggactat aaagatacca ggcgtttccc cctggaagct

7981 ccctcgtgcg ctctcctgtt ccgaccctgc cgcttaccgg atacctgtcc gcctttctcc

8041 cttcgggaag cgtggcgctt tctcatagct cacgctgtag gtatctcagt tcggtgtagg

8101 tcgttcgctc caagctgggc tgtgtgcacg aaccccccgt tcagcccgac cgctgcgcct

8161 tatccggtaa ctatcgtctt gagtccaacc cggtaagaca cgacttatcg ccactggcag

8221 cagccactgg taacaggatt agcagagcga ggtatgtagg cggtgctaca gagttcttga

8281 agtggtggcc taactacggc tacactagaa ggacagtatt tggtatctgc gctctgctga

8341 agccagttac cttcggaaaa agagttggta gctcttgatc cggcaaacaa accaccgctg

8401 gtagcggtgg tttttttgtt tgcaagcagc agattacgcg cagaaaaaaa ggatctcaag

8461 aagatccttt gatcttttct acggggtctg acgctcagtg gaacgaaaac tcacgttaag

8521 ggattttggt catgagatta tcaaaaagga tcttcaccta gatcctttta aattaaaaat

8581 gaagttttaa atcaatctaa agtatatatg agtaaacttg gtctgacagt taccaatgct

8641 taatcagtga ggcacctatc tcagcgatct gtctatttcg ttcatccata gttgcctgac

8701 tccccgtcgt gtagataact acgatacggg agggcttacc atctggcccc agtgctgcaa

8761 tgataccgcg agacccacgc tcaccggctc cagatttatc agcaataaac cagccagccg

8821 gaagggccga gcgcagaagt ggtcctgcaa ctttatccgc ctccatccag tctattaatt

8881 gttgccggga agctagagta agtagttcgc cagttaatag tttgcgcaac gttgttgcca

8941 ttgctacagg catcgtggtg tcacgctcgt cgtttggtat ggcttcattc agctccggtt

9001 cccaacgatc aaggcgagtt acatgatccc ccatgttgtg caaaaaagcg gttagctcct

9061 tcggtcctcc gatcgttgtc agaagtaagt tggccgcagt gttatcactc atggttatgg

9121 cagcactgca taattctctt actgtcatgc catccgtaag atgcttttct gtgactggtg

9181 agtactcaac caagtcattc tgagaatagt gtatgcggcg accgagttgc tcttgcccgg

9241 cgtcaatacg ggataatacc gcgccacata gcagaacttt aaaagtgctc atcattggaa

9301 aacgttcttc ggggcgaaaa ctctcaagga tcttaccgct gttgagatcc agttcgatgt

9361 aacccactcg tgcacccaac tgatcttcag catcttttac tttcaccagc gtttctgggt

9421 gagcaaaaac aggaaggcaa aatgccgcaa aaaagggaat aagggcgaca cggaaatgtt

9481 gaatactcat actcttcctt tttcaatatt attgaagcat ttatcagggt tattgtctca

9541 tgagcggata catatttgaa tgtatttaga aaaataaaca aataggggtt ccgcgcacat

9601 ttccccgaaa agtgccacac taaattgtaa gcgttaatat tttgttaaaa ttcgcgttaa

9661 atttttgtta aatcagctca ttttttaacc aataggccga aatcggcaaa atcccttata

9721 aatcaaaaga

//

Vector sequence G (Figure 5)

LOCUS Exported 9190 bp ds-DNA circular SYN

DEFINITION synthetic circular DNA

ACCESSION .

VERSION .

KEYWORDS .

SOURCE synthetic DNA construct

ORGANISM synthetic DNA construct

REFERENCE 1 (bases 1 to 9190)

AUTHORS Thomas Baier

FEATURES Location/Qualifiers

source 1..9190

/organism="synthetic DNA construct"

/mol_type="other DNA"

misc_feature 572..1334

/label=PSAD promoter

gene join(1347..1784,1930..2430,2576..3049,3195..3443)

/label=Patchoulol synthase

intron 1785..1929

/label=RBCS2 intron 1

intron 2431..2575

/label=RBCS2 intron 1

intron 3050..3194

/label=RBCS2 intron 1

CDS 3456..3473

/label=GSGSGS-Linker

gene join(3474..3672,3818..4134,4280..4477)

/label=mVenus

intron 3673..3817

/label=RBCS2 intron 1

intron 4135..4279

/label=RBCS2 intron 1

CDS 4478..4495

/codon_start=1

/label=GSGSGS-Linker

/translation="GSGSGS"

CDS join(4508..4526,4856..4857)

/codon_start=1

/label=GSGS-Linker

/translation="GSGSGSG"

intron 4527..4855

/label=RBCS2 intron 2

CDS 4858..4881

/codon_start=1

/product="peptide that binds Strep-Tactin(R), an engineered form of streptavidin"

/label=Strep-Tag II

/translation="WSHPQFEK"

misc_feature 4885..5118

/label=3'UTR

misc_feature 5119..5130

/label=MCS

promoter 5131..5397

/label=HSP70Ap

/label=HSP70Ap(1)

promoter join(5404..5632,5778..5779)

/label=RBCS2p promoter

/label=RBCS2p

intron 5633..5777

/label=RBCS2 intron 1

CDS 5820..6623

/codon_start=1

/label=APHVIII

/translation="MDDALRALRGRYPGCEWVVVEDGASGAGVYRLRGGGRELFVKVAALGAGVGLLGEAERLVWLAEVGIPVPRVVEGGGDERVAWLVTEAVPGRPASARWPREQRLDVAVALAGLARSLHALDWERCPFDRSLAVTVPQAARAVAEGSVDLEDLDEERKGWSGERLLAELERTRPADEDLAVCHGDLCPDNVLLDPRTCEVTGLIDVGRVGRADRHSDLALVLRELAHEEDPWFGPECSAAFLREYGRGWDGAVSEEKLAFYRLLDEFF"

misc_feature 6633..6871

/label=3'UTR

/label=3'UTR(1)

rep_origin complement(7331..7919)

/direction=LEFT

/label=ori

/note="high-copy-number ColE1/pMB1/pBR322/pUC origin of

replication"

CDS complement(8090..8950)

/codon_start=1

/gene="bla"

/product="beta-lactamase"

/label=AmpR

/note="confers resistance to ampicillin, carbenicillin, and related antibiotics"

/translation="MSIQHFRVALIPFFAAFCLPVFAHPETLVKVKDAEDQLGARVGYIELDLNSGKILESFRPEERFPMMSTFKVLLCGAVLSRIDAGQEQLGRRIHYSQNDLVEYSPVTEKHLTDGMTVRELCSAAITMSDNTAANLLLTTIGGPKELTAFLHNMGDHVTRLDRWEPELNEAIPNDERDTTMPVAMATTLRKLLTGELLTLASRQQLIDWMEADKVAGPLLRSALPAGWFIADKSGAGERGSRGIIAALGPDGKPSRIVVIYTTGSQATMDERNRQIAEIGASLIKHW"

promoter complement(8951..9055)

/gene="bla"

/label=AmpR promoter

rep_origin complement(join(9082..9190,1..347))

/direction=LEFT

/label=f1 ori

/note="f1 bacteriophage origin of replication; arrow

indicates direction of (+) strand synthesis"

ORIGIN

1 atagaccgag atagggttga gtgttgttcc agtttggaac aagagtccac tattaaagaa

61 cgtggactcc aacgtcaaag ggcgaaaaac cgtctatcag ggcgatggcc cactacgtga

121 accatcaccc taatcaagtt ttttggggtc gaggtgccgt aaagcactaa atcggaaccc

181 taaagggagc ccccgattta gagcttgacg gggaaagccg gcgaacgtgg cgagaaagga

241 agggaagaaa gcgaaaggag cgggcgctag ggcgctggca agtgtagcgg tcacgctgcg

301 cgtaaccacc acacccgccg cgcttaatgc gccgctacag ggcgcgtccc attcgccatt

361 caggctgcgc aactgttggg aagggcgatc ggtgcgggcc tcttcgctat tacgccagct

421 ggcgaaaggg ggatgtgctg caaggcgatt aagttgggta acgccagggt tttcccagtc

481 acgacgttgt aaaacgacgg ccagtgagcg cgcgtaatac gactcactat agggcgaatt

541 ggagctccac cgcggtggcg gccgctctag aaccaatcgt cacacgagcc ctcgtcagaa

601 acacgtctcc gccacgctct ccctctcacg gccgaccccg cagccctttt gccctttcct

661 aggccaccga caggacccag gcgctctcag catgcctcaa caacccgtac tcgtgccagc

721 ggtgcccttg tgctggtgat cgcttggaag cgcatgcgaa gacgaagggg cggagcaggc

781 ggcctggctg ttcgaagggc tcgccgccag ttcgggtgcc tttctccacg cgcgcctcca

841 cacctaccga tgcgtgaagg caggcaaatg ctcatgtttg cccgaactcg gagtccttaa

901 aaagccgctt cttgtcgtcg ttccgagaca tgttagcaga tcgcagtgcc acctttcctg

961 acgcgctcgg ccccatattc ggacgcaatt gtcatttgta gcacaattgg agcaaatctg

1021 gcgaggcagt aggcttttaa gttgcaaggc gagagagcaa agtgggacgc ggcgtgatta

1081 ttggtattta cgcgacggcc cggcgcgtta gcggcccttc ccccaggcca gggacgatta

1141 tgtatcaata ttgttgcgtt cgggcactcg tgcgagggct cctgcgggct ggggaggggg

1201 atctgggaat tggaggtacg accgagatgg cttgctcggg gggaggtttc ctcgccgagc

1261 aagccagggt taggtgttgc gctcttgact cgttgtgcat tctaggaccc cactgctact

1321 cacaacaagc ccatcatatg ggatccgagc tgtacgccca gagcgtgggc gtgggcgccg

1381 ccagccgccc cctggccaac ttccacccct gcgtgtgggg cgacaagttc atcgtgtaca

1441 acccccagag ctgccaggcc ggcgagcgcg aggaggccga ggagctgaag gtggagctga

1501 agcgcgagct gaaggaggcc agcgacaact acatgcgcca gctgaagatg gtggacgcca

1561 tccagcgcct gggcatcgac tacctgttcg tggaggacgt ggacgaggcc ctgaagaacc

1621 tgttcgagat gttcgacgcc ttctgcaaga acaaccacga catgcacgcc accgccctga

1681 gcttccgcct gctgcgccag cacggctacc gcgtgtcctg cgaggtgttc gagaagttca

1741 aggacggcaa ggacggcttc aaggtgccca acgaggacgg cgcggtgagt cgacgagcaa

1801 gcccggcgga tcaggcagcg tgcttgcaga tttgacttgc aacgcccgca ttgtgtcgac

1861 gaaggctttt ggctcctctg tcgctgtctc aagcagcatc taaccctgcg tcgccgtttc

1921 catttgcagg tggcggtgct ggagttcttc gaggccaccc acctgcgcgt gcacggcgag

1981 gacgtgctgg acaacgcctt cgacttcacc cgcaactacc tggagagcgt gtacgccacc

2041 ctgaacgacc ccaccgccaa gcaggtgcac aacgcgctga acgagttctc cttccgccgc

2101 ggcctgcccc gcgtggaggc ccgcaagtac atcagcatct acgagcagta cgccagccac

2161 cacaagggcc tgctgaagct ggccaagctg gacttcaacc tggtgcaggc gctgcaccgc

2221 cgcgagctgt ccgaggacag ccgctggtgg aagaccctgc aggtgcccac caagctgagc

2281 ttcgtgcgcg accgcctggt ggagagctac ttctgggcca gcggcagcta cttcgagccc

2341 aactacagcg tggcccgcat gatcctggcg aagggcctgg ccgtgctgag cctgatggac

2401 gacgtgtacg acgcctacgg caccttcgag gtgagtcgac gagcaagccc ggcggatcag

2461 gcagcgtgct tgcagatttg acttgcaacg cccgcattgt gtcgacgaag gcttttggct

2521 cctctgtcgc tgtctcaagc agcatctaac cctgcgtcgc cgtttccatt tgcaggagct

2581 gcagatgttc accgacgcga tcgagcgctg ggacgccagc tgcctggaca agctgcccga

2641 ctacatgaag atcgtgtaca aggccctgct ggacgtgttc gaggaggtgg acgaggagct

2701 gatcaagctg ggcgccccct accgcgccta ctacggcaag gaggccatga agtacgccgc

2761 ccgcgcctac atggaggagg cccagtggcg cgagcagaag cacaagccca ccaccaagga

2821 gtacatgaag ctggcgacca agacctgcgg ctacatcacc ctgatcatcc tgtcctgcct

2881 gggcgtggag gagggcatcg tgaccaagga ggcgttcgac tgggtgttca gccgcccgcc

2941 gttcatcgag gcgaccctga tcatcgcccg cctggtcaac gacatcaccg gccacgagtt

3001 cgagaagaag cgcgagcacg tgcgcaccgc cgtggagtgc tacatggagg tgagtcgacg

3061 agcaagcccg gcggatcagg cagcgtgctt gcagatttga cttgcaacgc ccgcattgtg

3121 tcgacgaagg cttttggctc ctctgtcgct gtctcaagca gcatctaacc ctgcgtcgcc

3181 gtttccattt gcaggagcac aaggtgggca agcaggaggt ggtgtccgag ttctacaacc

3241 agatggagag cgcctggaag gacatcaacg agggcttcct gcgccccgtg gagttcccca

3301 tccccctgct gtacctgatc ctgaacagcg tgcgcaccct ggaggtgatc tacaaggagg

3361 gcgacagcta cacccacgtg ggcccggcca tgcagaacat catcaagcag ctgtacctgc

3421 accccgtgcc ctacggcagc ggcagatctg acgtcggcag cggcagcggc agcgtgagca

3481 agggcgagga gctgttcacc ggcgtggtgc ccatcctggt ggagctggac ggcgacgtga

3541 acggccacaa gttcagcgtg agcggcgagg gcgagggcga cgccacctac ggcaagctga

3601 ccctgaagct gatctgcacc accggcaagc tgcccgtgcc ctggcccacc ctggtgacca

3661 ccctgggcta cggtgagtcg acgagcaagc ccggcggatc aggcagcgtg cttgcagatt

3721 tgacttgcaa cgcccgcatt gtgtcgacga aggcttttgg ctcctctgtc gctgtctcaa

3781 gcagcatcta accctgcgtc gccgtttcca tttgcaggcc tgcagtgctt cgcccgctac

3841 cccgaccaca tgaagcagca cgacttcttc aagagcgcca tgcccgaggg ctacgtgcag

3901 gagcgcacca tcttcttcaa ggacgacggt aactacaaga cccgcgccga ggtgaagttc

3961 gagggcgaca ccctggtgaa ccgcatcgag ctgaagggca tcgacttcaa ggaggacggc

4021 aacatcctgg gccacaagct ggagtacaac tacaacagcc acaacgtgta catcaccgcc

4081 gacaagcaga agaacggcat caaggccaac ttcaagatcc gccacaacat cgaggtgagt

4141 cgacgagcaa gcccggcgga tcaggcagcg tgcttgcaga tttgacttgc aacgcccgca

4201 ttgtgtcgac gaaggctttt ggctcctctg tcgctgtctc aagcagcatc taaccctgcg

4261 tcgccgtttc catttgcagg acggcggcgt gcagctggcc gaccactacc agcagaacac

4321 ccccatcggc gacggccccg tgctgctgcc cgacaaccac tacctgagct accagagcaa

4381 gctgagcaag gaccccaacg agaagcgcga ccacatggtg ctgctggagt tcgtgaccgc

4441 cgccggcatc accctgggca tggacgagct gtacaagggc agcggcagcg gcagcgatat

4501 cgaattcggc agcggcagcg gctcaggtga gcttgcgggg ttgcgagcaa cactccagca

4561 acgaacagtg cccaagtcag gaatctgcag tcagcctggg ctttcggcgg ctttttcttg

4621 ggcaaacagc ttgcactcat gccagcgcgg cttgtccagc ctcacttgag ctttccagct

4681 gctaccagcc gggctatacg acagcgacag agccatagcg tggaatcact tatttgggtt

4741 gccgaagtag cggtcggagc gtgagttctt ggtcaagccg ccccttatcc ggttcctgtc

4801 cgtgtctttg tccctcgttc acccttcgcg gcacccttca tccccttgct tgcaggttgg

4861 agccacccgc agttcgagaa gtaaccgctc cgtgtaaatg gaggcgctcg ttgatctgag

4921 ccttgccccc tgacgaacgg cggtggatgg aagatactgc tctcaagtgc tgaagcggta

4981 gcttagctcc ccgtttcgtg ctgatcagtc tttttcaaca cgtaaaaagc ggaggagttt

5041 tgcaattttg ttggttgtaa cgatcctccg ttgattttgg cctctttctc catgggcggg

5101 ctgggcgtat ttgaagcgac tagtacgcgt gctgaggctt gacatgattg gtgcgtatgt

5161 ttgtatgaag ctacaggact gatttggcgg gctatgaggg cgggggaagc tctggaaggg

5221 ccgcgatggg gcgcgcggcg tccagaaggc gccatacggc ccgctggcgg cacccatccg

5281 gtataaaagc ccgcgacccc gaacggtgac ctccactttc agcgacaaac gagcacttat

5341 acatacgcga ctattctgcc gctatacata accactcagc tagcttaaga tcccatccct

5401 agggcatgcc gggcgcgcca gaaggagcgc agccaaacca ggatgatgtt tgatggggta

5461 tttgagcact tgcaaccctt atccggaagc cccctggccc acaaaggcta ggcgccaatg

5521 caagcagttc gcatgcagcc cctggagcgg tgccctcctg ataaaccggc cagggggcct

5581 atgttcttta cttttttaca agagaagtca ctcaacatct taaaatggcc aggtgagtcg

5641 acgagcaagc ccggcggatc aggcagcgtg cttgcagatt tgacttgcaa cgcccgcatt

5701 gtgtcgacga aggcttttgg ctcctctgtc gctgtctcaa gcagcatcta accctgcgtc

5761 gccgtttcca tttgcaggaa gcttactccg ccctccccgg tgctgaagaa tttcgaagca

5821 tggacgatgc gttgcgtgca ctgcggggtc ggtatcccgg ttgtgagtgg gttgttgtgg

5881 aggatggggc ctcgggggct ggtgtttatc ggcttcgggg tggtgggcgg gagttgtttg

5941 tcaaggtggc agctctgggg gccggggtgg gcttgttggg tgaggctgag cggctggtgt

6001 ggttggcgga ggtggggatt cccgtacctc gtgttgtgga gggtggtggg gacgagaggg

6061 tcgcctggtt ggtcaccgaa gcggttccgg ggcgtccggc cagtgcgcgg tggccgcggg

6121 agcagcggct ggacgtggcg gtggcgctcg cggggctcgc tcgttcgctg cacgcgctgg

6181 actgggagcg gtgtccgttc gatcgcagtc tcgcggtgac ggtgccgcag gcggcccgtg

6241 ctgtcgctga agggagcgtc gacttggagg atctggacga ggagcggaag gggtggtcgg

6301 gggagcggct tctcgccgag ctggagcgga ctcggcctgc ggacgaggat ctggcggttt

6361 gccacggtga cctgtgcccg gacaacgtgc tgctcgaccc tcgtacctgc gaggtgaccg

6421 ggctgatcga cgtggggcgg gtcggccgtg cggaccggca ctccgatctc gcgctggtgc

6481 tgcgcgagct ggcccacgag gaggacccgt ggttcgggcc ggagtgttcc gcggcgttcc

6541 tgcgggagta cgggcgcggg tgggatgggg cggtatcgga ggaaaagctg gcgttttacc

6601 ggctgttgga cgagttcttc tgactcgagt gaccgctccg tgtaaatgga ggcgctcgtt

6661 gatctgagcc ttgccccctg acgaacggcg gtggatggaa gatactgctc tcaagtgctg

6721 aagcggtagc ttagctcccc gtttcgtgct gatcagtctt tttcaacacg taaaaagcgg

6781 aggagttttg caattttgtt ggttgtaacg atcctccgtt gattttggcc tctttctcca

6841 tgggcgggct gggcgtattt gaagcggacc cggtacccag cttttgttcc ctttagtgag

6901 ggttaattgc gcgcttggcg taatcatggt catagctgtt tcctgtgtga aattgttatc

6961 cgctcacaat tccacacaac atacgagccg gaagcataaa gtgtaaagcc tggggtgcct

7021 aatgagtgag ctaactcaca ttaattgcgt tgcgctcact gcccgctttc cagtcgggaa

7081 acctgtcgtg ccagctgcat taatgaatcg gccaacgcgc ggggagaggc ggtttgcgta

7141 ttgggcgctc ttccgcttcc tcgctcactg actcgctgcg ctcggtcgtt cggctgcggc

7201 gagcggtatc agctcactca aaggcggtaa tacggttatc cacagaatca ggggataacg

7261 caggaaagaa catgtgagca aaaggccagc aaaaggccag gaaccgtaaa aaggccgcgt

7321 tgctggcgtt tttccatagg ctccgccccc ctgacgagca tcacaaaaat cgacgctcaa

7381 gtcagaggtg gcgaaacccg acaggactat aaagatacca ggcgtttccc cctggaagct

7441 ccctcgtgcg ctctcctgtt ccgaccctgc cgcttaccgg atacctgtcc gcctttctcc

7501 cttcgggaag cgtggcgctt tctcatagct cacgctgtag gtatctcagt tcggtgtagg

7561 tcgttcgctc caagctgggc tgtgtgcacg aaccccccgt tcagcccgac cgctgcgcct

7621 tatccggtaa ctatcgtctt gagtccaacc cggtaagaca cgacttatcg ccactggcag

7681 cagccactgg taacaggatt agcagagcga ggtatgtagg cggtgctaca gagttcttga

7741 agtggtggcc taactacggc tacactagaa ggacagtatt tggtatctgc gctctgctga

7801 agccagttac cttcggaaaa agagttggta gctcttgatc cggcaaacaa accaccgctg

7861 gtagcggtgg tttttttgtt tgcaagcagc agattacgcg cagaaaaaaa ggatctcaag

7921 aagatccttt gatcttttct acggggtctg acgctcagtg gaacgaaaac tcacgttaag

7981 ggattttggt catgagatta tcaaaaagga tcttcaccta gatcctttta aattaaaaat

8041 gaagttttaa atcaatctaa agtatatatg agtaaacttg gtctgacagt taccaatgct

8101 taatcagtga ggcacctatc tcagcgatct gtctatttcg ttcatccata gttgcctgac

8161 tccccgtcgt gtagataact acgatacggg agggcttacc atctggcccc agtgctgcaa

8221 tgataccgcg agacccacgc tcaccggctc cagatttatc agcaataaac cagccagccg

8281 gaagggccga gcgcagaagt ggtcctgcaa ctttatccgc ctccatccag tctattaatt

8341 gttgccggga agctagagta agtagttcgc cagttaatag tttgcgcaac gttgttgcca

8401 ttgctacagg catcgtggtg tcacgctcgt cgtttggtat ggcttcattc agctccggtt

8461 cccaacgatc aaggcgagtt acatgatccc ccatgttgtg caaaaaagcg gttagctcct

8521 tcggtcctcc gatcgttgtc agaagtaagt tggccgcagt gttatcactc atggttatgg

8581 cagcactgca taattctctt actgtcatgc catccgtaag atgcttttct gtgactggtg

8641 agtactcaac caagtcattc tgagaatagt gtatgcggcg accgagttgc tcttgcccgg

8701 cgtcaatacg ggataatacc gcgccacata gcagaacttt aaaagtgctc atcattggaa

8761 aacgttcttc ggggcgaaaa ctctcaagga tcttaccgct gttgagatcc agttcgatgt

8821 aacccactcg tgcacccaac tgatcttcag catcttttac tttcaccagc gtttctgggt

8881 gagcaaaaac aggaaggcaa aatgccgcaa aaaagggaat aagggcgaca cggaaatgtt

8941 gaatactcat actcttcctt tttcaatatt attgaagcat ttatcagggt tattgtctca

9001 tgagcggata catatttgaa tgtatttaga aaaataaaca aataggggtt ccgcgcacat

9061 ttccccgaaa agtgccacac taaattgtaa gcgttaatat tttgttaaaa ttcgcgttaa

9121 atttttgtta aatcagctca ttttttaacc aataggccga aatcggcaaa atcccttata

9181 aatcaaaaga

//
